# Supplementary material for: Investigating the uptake, effectiveness and safety of COVID-19 vaccines: protocol for an observational study using linked UK national data
Source: BMJ Open. 2022 Feb 14;12(2):e050062. doi: 10.1136/bmjopen-2021-050062 (PMC8844955; doi:10.1136/bmjopen-2021-050062)

Supplementary Material

Appendix 1: Adverse Events Following Immunisation (AEFI) reported in pre-licensure COVID-19 vaccine trials

Adverse event	Pfizer-BioNTech (BNT162b2)	Oxford-AstraZeneca (AZD1222)	Moderna
Abdominal pain		Uncommon	
Acute peripheral facial paralysis (/palsy)	Rare (37 days after dose 1, 3-48 days after dose 2)		Rare (22-32 days after dose 2)
Anaphylaxis*	Not known		Not known
Arthralgia	Very common	Very common	Very common
Chills	Very common	Very common	Very common
Decreased appetite		Uncommon	
Dizziness		Uncommon	
Facial swelling			Rare (1-2 days post-vaccination in vaccinees with history of injection of dermatological fillers)
Fatigue	Very common	Very common	Very common
Headache	Very common	Very common	Very common
Hyperhidrosis		Uncommon	
Hypersensitivity	Not known		Not known
Influenza-like illness		Common	
Injection site bruising**		Very common	
Injection site erythema		Very common	Common
Injection site induration		Common	
Injection site pain	Very common	Very common	Very common
Injection site pruritus	Uncommon	Very common	Uncommon
Injection site rash			Common
Injection site redness	Common		
Injection site swelling	Very common	Very common	Very common
Injection site tenderness		Very common	
Injection site urticaria			Common
Injection site warmth		Very common	
Insomnia	Uncommon		
Lymphadenopathy***	Uncommon	Uncommon	Very common
Malaise	Uncommon	Very common	
Myalgia	Very common	Very common	Very common
Nausea****	Common	Very common	Very common
Pain in extremity	Uncommon		
Pruritus		Uncommon	
Pyrexia*****	Very common	Very common	Very common
Rash		Uncommon	Common
Vomiting		Common	
* Anaphylaxis reported in post-marketing setting for Moderna vaccine.			

Vasileiou E, *et al. BMJ Open* 2022; 12:e050062. doi: 10.1136/bmjopen-2021-050062

** Injection site bruising includes injections site haematoma
*** Lymphadenopathy captured as axillary lymphadenopathy on the same side as the injection site for Moderna vaccine.
**** A higher frequency of pyrexia observed after dose 2 for the Pfizer BioNTech vaccine.
***** Pyrexia includes feverishness
Note. Very common (≥1/10), common (≥1/100 to <1/10), uncommon (≥1/1,000 to <1/100), rare (≥1/10,000 to <1/1,000), very rare (<1/10,000), not known (cannot be estimated).

Very rare events of neuroinflammatory disorders have been reported following vaccination with the Oxford AstraZeneca vaccine, but a causal relationship has not been established.

Appendix 2: List of AEFI for influenza vaccines

- Anaphylactic reactions
- Arthropathy
- Bell's palsy
- Conjunctivitis
- Coryza
- Cough
- Decreased appetite
- Diarrhoea
- Drowsiness
- Epistaxis
- Facial oedema
- Fatigue
- Fever / pyrexia
- Guillain-Barré syndrome
- Headache
- Hoarseness
- Hypersensitivity reactions
- Irritability
- Local symptoms (i.e. local erythema)
- Malaise
- Muscle aches / myalgia
- Nasal congestion
- Nausea
- Oropharyngeal pain
- Peripheral tremor
- Rash
- Rhinorrhoea
- Seizure / febrile convulsions
- Vomiting
- Wheezing

Appendix 3: Schematic presentation of the self-controlled case series study design

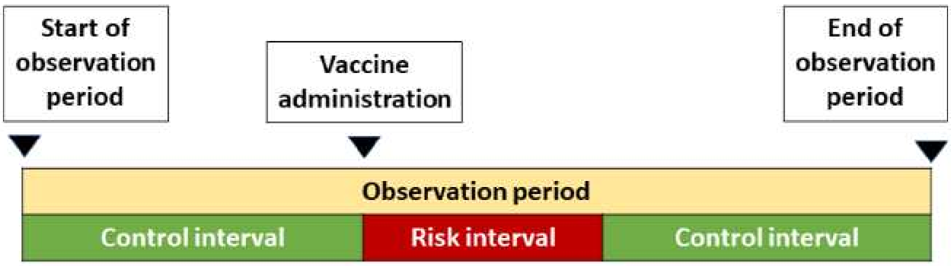

Supplement: Supplementary data [file bmjopen-2021-050062supp001.pdf]
