# Supplementary material for: Investigating the uptake, effectiveness and safety of COVID-19 vaccines: protocol for an observational study using linked UK national data
Source: BMJ Open. 2022 Feb 14;12(2):e050062. doi: 10.1136/bmjopen-2021-050062 (PMC8844955; doi:10.1136/bmjopen-2021-050062)
Supplement: Author's manuscript [file bmjopen-2021-050062.draft_revisions.pdf]

BMJ Open is committed to open peer review. As part of this commitment we make the peer review history of every article we publish publicly available.

When an article is published we post the peer reviewers' comments and the authors' responses online. We also post the versions of the paper that were used during peer review. These are the versions that the peer review comments apply to.

The versions of the paper that follow are the versions that were submitted during the peer review process. They are not the versions of record or the final published versions. They should not be cited or distributed as the published version of this manuscript.

BMJ Open is an open access journal and the full, final, typeset and author-corrected version of record of the manuscript is available on our site with no access controls, subscription charges or pay-per-view fees (http://bmjopen.bmj.com).

If you have any questions on BMJ Open's open peer review process please email info.bmjopen@bmj.com

BMJ Open

Investigating the uptake, effectiveness and safety of COVID-19 vaccines: protocol for an observational study using linked UK national data

Journal:	*BMJ Open*
Manuscript ID	bmjopen-2021-050062
Article Type:	Protocol
Date Submitted by the Author:	09-Feb-2021

Complete List of Authors:	Vasileiou, Eleftheria; The University of Edinburgh, Usher Institute<br>Shi, Ting; The University of Edinburgh, Usher Institute<br>Kerr, Steven; The University of Edinburgh, Usher Institute<br>Robertson, Chris; University of Strathclyde, Department of Mathematics and Statistics; Public Health Scotland Glasgow Office<br>Joy, Mark; University of Oxford, Nuffield Department of Primary Care Health Sciences<br>Tsang, Ruby; University of Oxford, Nuffield Department of Primary Care Health Sciences<br>McGagh, Dylan; University of Oxford, Nuffield Department of Primary Care Health Sciences<br>Williams, John; University of Oxford, Nuffield Department of Primary Care Health Sciences<br>Hobbs, Richard; University of Oxford, Nuffield Department of Primary Care Health Sciences<br>de Lusignan, Simon; University of Oxford, Nuffield Department of Primary Care Health Sciences<br>Bradley, Declan; Queen's University Belfast, School of Medicine, Dentistry and Biomedical Sciences<br>OReilly, Dermot; Queen's University Belfast, School of Medicine, Dentistry and Biomedical Sciences<br>Murphy, Siobhan ; Queen's University Belfast, School of Medicine, Dentistry and Biomedical Sciences<br>Chuter, Antony; BREATHE – The Health Data Research Hub for Respiratory Health<br>Beggs, Jillian; BREATHE – The Health Data Research Hub for Respiratory Health<br>Ford, David; Swansea University Medical School, Population Data Science<br>Orton, Chris; Swansea University Medical School, Population Data Science<br>Akbari, Ashley; Swansea University Medical School, Population Data Science<br>Bedston, Stuart; Swansea University Medical School, Population Data Science<br>Davies, Gareth; Swansea University Medical School, Population Data Science<br>Griffiths, Rowena; Swansea University Medical School, Population Data Science<br>Lyons, Jane; Swansea University Medical School, Population Data

Science
Lyons, Ronan; Swansea University Medical School, Population Data Science
North, Laura; Swansea University Medical School, Population Data Science
Perry, Malorie; Public Health Wales, Vaccine Preventable Disease Programme
Torabi, Fatemeh; Swansea University Medical School, Population Data Science
Pickett, James; Health Data Research UK
McMenamin, Jim; Public Health Scotland Glasgow Office
McCowan, Colin ; University of St Andrews, School of Medicine
Agrawal, Utkarsh; University of St Andrews, School of Medicine
Wood, Rachael; Public Health Scotland; The University of Edinburgh, Usher Institute
Stock, Sarah; The University of Edinburgh Usher Institute of Population Health Sciences and Informatics, Centre for Medical Informatics; Public Health Scotland,
Moore, Emily; Public Health Scotland, Data Driven Innovation
Henery, Paul; University of Glasgow, MRC/CSO Social and Public Health Sciences Unit
Simpson, Colin; Victoria University of Wellington, Wellington School of Health, Faculty of Health; The University of Edinburgh, Usher Institute
Sheikh, Aziz; The University of Edinburgh, Usher Institute

Keywords: COVID-19, Respiratory infections < THORACIC MEDICINE, Epidemiology < INFECTIOUS DISEASES, Public health < INFECTIOUS DISEASES

SCHOLARONE™
Manuscripts

Investigating the uptake, effectiveness and safety of COVID-19 vaccines: protocol for an observational study using linked UK national data

Dr Eleftheria Vasileiou, Usher Institute, The University of Edinburgh, NINE Edinburgh BioQuarter, Edinburgh, UK, EH16 4UX, eleftheria.vasileiou@ed.ac.uk, +44 07732961139 (corresponding author)

Dr Ting Shi, Usher Institute, The University of Edinburgh, Edinburgh, UK

Dr Steven Kerr, Usher Institute, The University of Edinburgh, Edinburgh, UK

Prof Chris Robertson, Department of Mathematics and Statistics, University of Strathclyde, Glasgow, UK and Public Health Scotland, Glasgow, UK

Dr Mark Joy, Nuffield Department of Primary Care Health Sciences, University of Oxford, Oxford, UK

Dr Ruby Tsang, Nuffield Department of Primary Care Health Sciences, University of Oxford, Oxford, UK

Dylan McGagh, Nuffield Department of Primary Care Health Sciences, University of Oxford, Oxford, UK

John Williams, Nuffield Department of Primary Care Health Sciences, University of Oxford, Oxford, UK

Prof Richard Hobbs, Nuffield Department of Primary Care Health Sciences, University of Oxford, Oxford, UK

Prof Simon de Lusignan, Nuffield Department of Primary Care Health Sciences, University of Oxford, Oxford, UK

Prof Declan Bradley, School of Medicine, Dentistry and Biomedical Sciences, Queen's University Belfast, Belfast, UK

Prof Dermot O'Reilly, School of Medicine, Dentistry and Biomedical Sciences, Queen's University Belfast, Belfast, UK

Dr Siobhan Murphy, School of Medicine, Dentistry and Biomedical Sciences, Queen's University Belfast, Belfast, UK

Antony Chuter, PPIE Lead, BREATHE – The Health Data Research Hub for Respiratory Health, UK

Jillian Beggs, PPIE Lead, BREATHE – The Health Data Research Hub for Respiratory Health, UK

Prof David Ford, Population Data Science, Swansea University Medical School, Swansea, UK

Chris Orton, Population Data Science, Swansea University Medical School, Swansea, UK

For peer review only - http://bmjopen.bmj.com/site/about/guidelines.xhtml

Ashley Akbari, Population Data Science, Swansea University Medical School, Swansea, UK

Dr Stuart Bedston, Population Data Science, Swansea University Medical School, UK

Gareth Davies, Population Data Science, Swansea University Medical School, UK

Rowena Griffiths, Population Data Science, Swansea University Medical School, UK

Jane Lyons, Population Data Science, Swansea University Medical School, UK

Prof Ronan A Lyons, Population Data Science, Swansea University Medical School, UK

Laura North, Population Data Science, Swansea University Medical School, UK

Malorie Perry, Vaccine Preventable Disease Programme, Public Health Wales, UK

Fatemeh Torabi, Population Data Science, Swansea University Medical School, UK

Dr James Pickett, Health Data Research UK, London, UK

Dr Jim McMenamin, Public Health Scotland, Glasgow, UK

Prof Colin McCowan, School of Medicine, University of St. Andrews, St Andrews, UK

Dr Utkarsh Agrawal, School of Medicine, University of St. Andrews, St Andrews, UK

Dr Rachael Wood, Usher Institute, The University of Edinburgh, Edinburgh, UK, Public Health Scotland, Edinburgh, UK and Child Life and Health, University of Edinburgh, Edinburgh, UK

Dr Sarah Jane Stock, Tommy's Centre for Maternal and Fetal Health, The University of Edinburgh MRC Centre for Reproductive Health, Edinburgh, UK and Usher Institute, The University of Edinburgh, Edinburgh, UK

Dr Emily Moore, Public Health Scotland, Glasgow, UK

Paul Henery, MRC/CSO Social and Public Health Sciences Unit, University of Glasgow, Glasgow, UK

Prof Colin R Simpson, Wellington School of Health, Faculty of Health, Victoria University of Wellington, Wellington, New Zealand, Usher Institute, The University of Edinburgh, Edinburgh, UK

Prof Aziz Sheikh, Usher Institute, The University of Edinburgh, Edinburgh, UK

Keywords: COVID-19, coronavirus, vaccine, safety, effectiveness, uptake

For peer review only - http://bmjopen.bmj.com/site/about/guidelines.xhtml

ABSTRACT

Introduction

The novel coronavirus SARS-CoV-2, which emerged in December 2019 has caused millions of deaths and severe illness worldwide. Numerous vaccines are currently under development of which a few have now been authorised for population level administration in some countries. By the end of January 2021, the first doses of vaccines which have been approved by the UK's Medicines and Healthcare products Regulatory Agency (MHRA) had been administered to over 10 million people across the UK. We aim to assess the uptake rates, effectiveness, and safety of all currently approved COVID-19 vaccines in the UK.

Methods and analysis

We will use prospective cohort study designs to assess vaccine uptake and effectiveness against clinical outcomes and deaths. Test-negative case-control study design will be used to assess vaccine effectiveness (VE) against laboratory confirmed SARS-CoV-2 infection. Self-controlled case series and retrospective cohort study designs will be carried out to assess vaccine safety against mild-to-moderate and severe adverse events, respectively. Individual level pseudonymised data from primary care, secondary care, laboratory test and death records will be linked and analysed in secure research environments in each UK nation. Univariate and multivariate logistic regression models will be carried out to estimate vaccine uptake levels in relation to various population characteristics. VE estimates on against laboratory confirmed SARS-CoV-2 infection will be generated using a generalised additive logistic model. Time-dependent Cox models will be used to estimate the vaccine effectiveness against clinical outcomes and deaths. The safety of the vaccines will be assessed using logistic regression models with an offset for the length of the risk period. Where possible, data will be meta-analysed across the UK nations.

Ethics and dissemination

Approvals have been obtained from relevant ethics bodies in each UK nation. Findings will be made available to national policymakers, presented at conferences and published in peer-reviewed journals.

For peer review only - http://bmjopen.bmj.com/site/about/guidelines.xhtml

Strengths and limitations of this study

- We will use national data for each UK nation and across the UK general population.
- Rapid and robust real-time estimates on uptake, effectiveness and safety of COVID-19 vaccines will be provided using data from existing national pandemic platforms in the UK. To do this, individual-level national data from general practices, hospitals, death registry and laboratory tests will be linked and analysed in secure research environments available in each UK nation.
- This is an observational study and analyses are therefore potentially susceptible to residual or unmeasured confounders.

For peer review only

INTRODUCTION

The first human cases infected by the novel coronavirus Severe Acute Respiratory Syndrome Coronavirus 2 (SARS-CoV-2) pathogen were detected in Wuhan, China in December 2019.[1] On 11 March 2020, the World Health Organization (WHO) declared the coronavirus 2019 disease (COVID-19) as a global pandemic, which as of 07 February 2021 has caused more than 105 million infections and two million deaths worldwide.[1] The need for vaccines against this novel virus triggered an emergency response by governments, pharmaceutical companies, and research institutions to develop, licence and manufacture COVID-19 vaccines at scale. Dozens of COVID-19 vaccines are currently under development with some vaccines now having successfully completed their pre-licensure clinical trials and approved for population vaccine administration.[2,3] The speed with which the world's first vaccines [3] were available for mass administration at the end of 2020 is unprecedented given that typically it takes years for a vaccine to be available for use at a population level.[4]

The Pfizer-BioNTech and Oxford-AstraZeneca were amongst the first vaccines approved by national regulatory authorities such as the United Kingdom's (UK) Medicines and Healthcare products Regulatory Agency (MHRA).[5] Based on pre-licensure clinical trials, the Pfizer-BioNTech vaccine was 95% effective at preventing laboratory-confirmed COVID-19 illness in individuals without evidence of previous infection.[6] The Oxford-AstraZeneca vaccine has also reported significant efficacy of 64% and 70% after one and two doses, respectively against symptomatic disease.[7] Both vaccines were well tolerated with mild-to-moderate side-effects mostly reported.[6,7] As of 06 February 2021, over 12 million and 511,447 people across the UK have received their first and second vaccine doses respectively.[8] As of the 8 January 2021, the UK has also approved a third COVID-19 vaccine manufactured by Moderna which has shown 94% efficacy against confirmed SARS-CoV-2 infection after receipt of second dose.[9] At the time of writing, the Moderna vaccine is not in use in the UK.[10]

Timely post-licensure monitoring of coverage, protection and safety of these newly introduced vaccines is imperative.[11] Specifically, robust observational epidemiological studies are required to measure coverage rates in the population in relation to demographic and other population characteristics, assess protection levels against infection, severe illness and deaths, and to detect adverse events. These post-marketing observational studies will add additional value to the pre-licensure clinical trials as they can assess real-life effects of the COVID-19 vaccines and the impact of the vaccination programme at population levels.[12] All UK licensed vaccines have demonstrated high efficacy; however, more evidence is needed about the type, level, and duration of protection for different segments of the population. The recommended time period between administration of the first and second doses of the Pfizer-BioNTech [6] and Oxford-AstraZeneca [7] vaccines are 3 and 6 weeks, respectively. The UK government has recently decided to lengthen this gap to up to 12 weeks for both vaccines.[13] This is because of the desire to provide some degree of protection to as many people as possible

For peer review only - http://bmjopen.bmj.com/site/about/guidelines.xhtml

and the decision is supported by findings from the Oxford-AstraZeneca vaccine trial, which have shown that vaccine efficacy was higher (65%) in the group that were administered the second dose more than six weeks after the first dose, compared to the group given the second dose less than six weeks after the first dose (53%).[7,14] A simulation study has also shown that high vaccine coverage even with less efficacious vaccines (due to partial immunisation) can lead to a greater reduction of SARS-CoV-2 infection levels compared to a lower vaccine coverage with more efficacious vaccines.[15] On the other hand, some experts have expressed their concerns that delay between doses could increase the risk of vaccine-resistant strains emerging due to a partially immunised population.[14] The provision of timely estimates on the protection conferred between doses is thus urgently needed. The assessment of vaccine-induced adverse events also needs to be carried out, particularly for rare adverse events that are usually only detectable in large population studies.[16] The rate of vaccine receipt by demographic, socioeconomic and other epidemiological characteristics also needs to be measured. A recent United States (US) study surveyed 1678 adults and over a quarter of them (27%) stated that they probably or definitely would not get a COVID-19 vaccine.[17] Highest vaccine hesitancy was found in those aged 30-49 years (36%), rural residents (35%), Black adults (35%), essential workers (33%), health care workers (29%), male adults (29%) and ages 18-29 years (28%).[17] This is particularly concerning given that Black adults and health care workers are some of the subgroups of the population that have been most at risk in this pandemic.[17] The identification of key factors related to vaccine uptake may be useful in efforts to increase uptake and subsequently maximise the impact of the COVID-19 vaccination programme in the UK.

The aims of this study are to assess the uptake, effectiveness, and safety of the currently licenced COVID-19 vaccines (Pfizer-BioNTech, Oxford-AstraZeneca and Moderna) in the UK. We will also seek to assess any additional licensed vaccines during the course of this study. Specifically, our objectives are to: measure variation in vaccine uptake in relation to population characteristics; b) assess vaccine effectiveness (VE) against infection, transmission, [18] severe outcomes, and deaths; and c) identify the risk of adverse events following immunisation (AEFIs).

METHODS

Study design and population

A prospective cohort study design will be used to measure variations in vaccine uptake and assess VE against severe illness, deaths, secondary SARS-CoV-2 infection due to household transmission. A test-negative design (TND) case-control study will be carried out to assess VE against laboratory confirmed COVID-19 infection. In the TND, cases and controls will be those with a positive and negative test for COVID-19, respectively.[19]A self-controlled case series (SCCS) study design will be used to assess the risk of AEFI. The SCCS study will be used to determine the relative incidence of adverse events for exposed time periods (periods following vaccine administration) compared to unexposed time periods (pre- or post-vaccination periods unrelated to vaccination) in individuals who present with the outcome of interest.[20] For more severe and event dependent safety outcomes, a retrospective cohort study will be considered.

For peer review only - http://bmjopen.bmj.com/site/about/guidelines.xhtml

Overview of linked databases

We will use pseudonymised individual level data routinely collected at primary, secondary, linked with mortality, laboratory and vaccination data across the UK. Unique national datasets will be developed and hosted within secure research environments in each UK nation with standardised individual-level analyses run across datasets. Pooled estimates across the UK national will also be calculated. For England, data from the Royal College of General Practitioners Research and Surveillance Centre (RCGP RSC) (approximately 5.4 million people) [21] will be used. We will also access and analyse national coverage data from Northern Ireland, where we estimate an approximately 30% population coverage. For Scotland, data will be derived from the Early Pandemic Evaluation and Enhanced Surveillance of COVID-19 (EAVE II) (approximately 5.3 million people).[22] For Wales, data from the Controlling COVID19 through enhanced population surveillance and intervention (ConCOV) (approximately 3.2 million people) [23,24] and the Secure Anonymised Information Linkage (SAIL) Databank [25] will be used. See Table 1 for details on data sources from each UK nation.

English primary care data will be held in the Oxford-Royal College of General Practitioners Clinical Informatics Digital Hub (ORCHID).[26] Data will be pseudonymised as close to source as possible using an NHS Digital Data Security and Protection (DSP) approved method.[27,28] NHS Digital will link additional data to these pseudonyms.

For the Northern Irish data, the Honest Broker Service will carry out the data linkage and offer a secure environment for researchers to access and analyse the pseudonymised individual level data. The Health Care Number (HCN) will be used to link individuals' data though replaced with an anonymous study ID in analysis dataset.

For the Scottish data, Public Health Scotland (PHS) will carry out the data linkage and offer a secure environment for researchers to access and analyse the pseudonymised individual level data.[22] The Community Health Index (CHI) number (a unique identifier for each resident receiving healthcare) will be used to link individuals' data which will be replaced by a study ID.

Access to relevant Welsh data will occur within the Secure Anonymised Information Linkage (SAIL) Databank TRE,[25] in collaboration with the ConCOV project.[23,24] Data will be linked and anonymised from NHS sources via a mature split-file system. All identifiable data will stay within the NHS, and will be linked to the Welsh Demographic Service, pseudonymised, and encrypted by the NHS Wales Informatics Service, with pseudonymised demographics supplied to the SAIL system at Swansea University,[25] where the non-identifiable clinical data are held. SAIL will link the demographic data to relevant clinical data, and then further encrypt the linked data before presenting to research teams within a secure virtual desktop.

Exposure (vaccination) data

For peer review only - http://bmjopen.bmj.com/site/about/guidelines.xhtml

In England, data on vaccination will derive from GPs and the National Immunisation Management Service (NIMS).[29] NIMS is the System of Record for the NHS COVID-19 vaccination programme in England developed by NHS Digital.[29] NIMS will collect any demographic, GP and employee (for NHS) data to identify groups of the population eligible for vaccination. Data collected from NIMS will also feed back into GP systems so that an individual's electronic health record is updated regarding to their vaccination history.[29] Vaccination data in GP records will be recorded using the Systematised Nomenclature of Medicine (SNOMED) Clinical Terms (CT).[30] RCGP RSC now uses SNOMED CT for all its key variables including vaccine data.[31] A key part of these curated variables are those for COVID-19, there have been three iterations of these and we have carefully curated case definitions.[32-34]

In Northern Ireland, GP practices and Trust vaccination sites are currently delivering the COVID-19 vaccination programme. Vaccination data from GP practices and Trust vaccination sites will be stored in the Vaccination Management System (VMS) and made available via the Honest Broker Service (HBS) secure research environment.[35]

In Scotland, General Practitioners (GPs) usually facilitate vaccination programmes and record any data related to vaccine administration. GPs use Read codes to code and record relevant information arising from a patient consultation.[22] Additional data on vaccination will also be available via the Turas Vaccination Management Tool (TVMT), which is a web-based tool that enables frontline vaccinators to capture and create real-time patient vaccination records. PHS is currently collating vaccination data from Turas.[36] Scheduled vaccinations for children may also be recorded in the Scottish Immunisation Recall System (SIRS) database. Data on vaccinations administered at schools and not in GPs may thus derive from SIRS.[22]

In Wales, the vaccine programme is administered and recorded nationally in the all Wales Immunisation System (WIS) and is available in SAIL through the COVID Vaccine Data (CVVD). This is a separate independent system and data source to the GP data, which is also available in SAIL with vaccination recorded using Read codes.[25]

Outcome data

Laboratory confirmed outcomes

For England, most (Pillar 2) community testing is in the GP record, though for this study we will link to resources held by NHS Digital.[37]

For Northern Ireland, the Pillar 1 dataset is extracted from the Laboratory Information Systems in Northern Ireland hospitals on a daily basis.[37] It contains details of COVID-19 antigen tests carried out in each of the hospital laboratories, including those processed on behalf of primary care, social care and community settings. Pillar 2 data is processed by NHS Digital and extracts for NI residents are sent to the NI Public Health Agency.[37] Data is are routinely extracted from operational systems across the Health and Social Care (HSC) and loaded into the regional data warehouse by the HSC Business Services Organisation.

For peer review only - http://bmjopen.bmj.com/site/about/guidelines.xhtml

For Scotland, laboratory results from Scottish diagnostic and reference laboratories are captured by the Electronic Communication of Surveillance in Scotland (ECOSS) database, which can be used for surveillance and research purposes.[22] Laboratory results from NHS and community (Lighthouse laboratory) testing centres will also be available through ECOSS.

For Wales, national coverage of Pillar 1, 2 and 3 data from all NHS and private laboratories will be available, as well as national lateral flow testing data.[37]

We will also pursue to access genome sequencing data in a proportion of laboratory tests positive for SARS-CoV-2 available from national sequencing centres where possible.

Clinical outcomes

Data on primary care consultations for COVID-19 illness will be accessed via GP electronic health records in each UK nation.

In England, we will link to secondary care data through collections held by NHS Digital. Hospital data are held in two forms: Hospital Episode Statistics (HES), which are the long-term validated record; and Secondary Uses Services (SUS) which is an extract of contemporary operational data which after validation will become HES. We plan to use HES and also have an extract of intensive care data. We will access these data through NHS Digital's Data Access Request Service (DARS).[38] We also have SARS-CoV-2 virology data that were collected through the sentinel surveillance system.[39] The English primary care sentinel system within RCGP RSC has a strong working relationship with Public Health England (PHE), with whom we have worked closely for over half a century.[40]

In Northern Ireland, the Admissions and Discharge dataset will be used to collate data relating to admitted patient care delivered by NHS hospitals in Northern Ireland, generated by the patient administration systems within each hospital.[41]

In Scotland, data on patients receiving care in general or acute hospitals are recorded in the Scottish Morbidity Record 01 (SMR01).[22] The International Statistical Classification of Diseases and Related Health Problems, Tenth Revision (ICD-10) codes are used to index any diagnoses recorded in the patient's medical notes by a clinician.[22] Consistent and high level (>90%) of data accuracy have been shown in recent data quality reports for the SMR01 database.[20] Data on adult patients admitted to general intensive care units will derive from the Scottish Intensive Care Society Audit Group (SICSAG) database.[22]

In Wales, data on all interactions with secondary care including Accident and Emergency events (Emergency Department Dataset – EDDS), inpatient hospital admissions (Patient Episode Database for Wales - PEDW), intensive care (Critical Care DataSet – CDDS and Intensive Care National Audit & Research Centre - ICNARC) and outpatient appointments and activity (Outpatient Database for Wales – OPDW) are all available within SAIL Databank.[25] There are also a collection of specialised services and condition specific secondary care such as cancer which are available within SAIL.[25]

For peer review only - http://bmjopen.bmj.com/site/about/guidelines.xhtml

Deaths

In England, NHS Digital's Personal Demographic Service flags the date of death in the GP record. We have previously used these data of death to report excess mortality, both overall [42,43] and in people with known COVID-19 status.[44] We will augment these data with the certificated cause of death provided by the Office for National Statistics (ONS), which will be linked for us at individual pseudonymised patient level by NHS Digital.

In Northern Ireland, the National Health Applications and Infrastructure Services (NHAIS) will be used for mortality data.[45] NHAIS receives regular updates from GRO (General Register Office) on fact and cause of death.[45]

In Scotland, the death registry within National Records of Scotland (NRS) records information included in the death certificates.[22] ICD-10 codes on deaths records derive from diagnoses recorded by the certifying doctor on the death certificate.[22]

In Wales, multiple sources of mortality data including information from the National Population Spine (Welsh Demographic Service – WDSD), ONS death data (ADDE and ADDD) and a national NHS master patient index record (Consolidated Death Data Source – CDDS) will be accessed to retrieve complete, harmonised details on cause and associated mortality details.[25]

Pregnancy and neonatal outcomes

Capturing vaccine exposure in pregnancy is important.[46] As no trial to date has included pregnant women this type of study is the only opportunity to explore safety and effectiveness in pregnant women and their babies.

In England, we will use a customised "sliding window" to capture pregnancy data.[47]

In Northern Ireland, data will be accessed via the Northern Ireland Maternity System (NIMATS).[48]

In Scotland, pregnancy and neonatal outcomes in Scottish participants will be identified through the COVID-19 in Pregnancy in Scotland (COPS) study.[49]

Data sources for Wales will be sought via the SAIL Databank, replicating the approach in England.[25]

Exposure definitions

Data on the currently licenced COVID-19 vaccines, including Pfizer-BioNTech, Oxford-AstraZeneca and Moderna, will be derived from GPs, National Immunisation Management Service (NIMS), TVMT/PHS, WIS and HSC Trusts databases. For the Pfizer-BioNTech, the time period from 1 December 2020 to day 9 post-vaccination will be defined as 'unvaccinated or unexposed' and the period from day 10 post-vaccination until the end of last day of this cohort will be defined as 'vaccinated or exposed'.[6] For the Oxford-AstraZeneca, an

For peer review only - http://bmjopen.bmj.com/site/about/guidelines.xhtml

'unvaccinated or unexposed' period will be taken from 1 December to day 20 post-vaccination and a 'vaccinated or exposed' period will be taken from day 21 post-vaccination.[7] Based on results from the pre-licensure clinical trials, it is unclear how long it takes for an individual to be fully protected post-vaccination. We will also examine how VE levels change for cohorts defined by different time periods (e.g., 7, 14, 21 and 28 days post-vaccination). Vaccinated and unvaccinated periods for the Moderna vaccine will be determined once results from phase 3 clinical trials on vaccine efficacy are available.[50]

Outcome definitions

Adverse events following immunisation (AEFI): Adverse events to be monitored are derived from the safety results of the pre-licensure vaccine clinical trials, common side-effects related to influenza vaccines and an unpublished study protocol of an ongoing observational study.[6,7,51,52] These include use of health services such as GP or out-of-hours GP consultation, accident and emergency (A&E) department attendance, inpatient hospital admission and admission to intensive care units (ICU) for suspected adverse events. Safety of vaccines in pregnant women will also be considered once vaccines are widely administered in this group of the population. A full list of candidate AEFI is available in in the Supplementary Material, Appendix 1-2.

Vaccine effectiveness outcomes: Protective effects of the vaccines will be assessed against the following outcomes: a) RT-PCR laboratory confirmed COVID-19 infection; b) GP consultations related to suspected or confirmed COVID-19 illness; c) A&E attendance related to suspected or confirmed COVID-19 illness at presentation; d) hospital admissions related to confirmed COVID-19 illness; e) ICU admissions related to confirmed COVID-19 illness; and f) deaths related to suspected or confirmed COVID-19 illness. We will also explore the effects of vaccines on secondary SARS-CoV-2 infection due to household transmission. VE against pregnancy and neonatal outcomes will be explored once the vaccines are more widely available to pregnant women.

Population characteristics, confounding factors, and effect modifiers

A number of key characteristics that could explain variation in vaccine uptake will be considered. In addition, these characteristics could confound our planned analyses. We will determine and include these characteristics or potential confounders at the baseline of our study's cohort which include (see Table 1 for details on data sources for each UK nation):

Socio-demographics: Sex at birth will be included in a binary format (females and males). Age will be included in bands that will be determined based on available vaccination data. Socio-economic status will be assessed by the following national versions of area level deprivation indices: a) Index of Multiple Deprivation (IMD), 2019 version for England [53,54]; b) the Scottish Index of Multiple Deprivation (SIMD),[22]; c) the Welsh Index of Multiple Deprivation (WIMD) [55] and d) the Northern Ireland Multiple Deprivation Measure (NIMDM).[56] For England, we will maximise the capture of ethnicity data through the use of a customised ontology.[57] Ethnicity data for the other UK nations will also be included if available.

For peer review only - http://bmjopen.bmj.com/site/about/guidelines.xhtml

Geographic: In England, the RSC will use the ONS data on population density and classify households as rural, urban (town and city), or conurbation.[58] The RSC also has a unique household key, so we can report household incidence of respiratory and other infectious conditions.[59] In Scotland, settlement type will be included using the urban/rural 6-fold classification (UR6).[22] In Wales, urban and rural household classification that is based on the Lower-layer Super Output Area (LSOA) of the person's residence information will be used.[24] In Northern Ireland, NISRA's Statistical Classification and Delineation of Settlements will be used to determine settlement type.[60] Type of residence will also be considered such as private residence, care home and social/council housing for each UK nation if data are available.

Smoking status: Smoking status will be included through four categories: smoker, ex-smoker, non-smoker and 'not recorded'.

At-risk underlying medical conditions: Based on the QCOVID algorithm [61] we will consider the following conditions: a) cardiovascular conditions (atrial fibrillation, heart failure, stroke, peripheral vascular disease, coronary heart disease, congenital heart disease); b) diabetes (type 1 and type 2); c) respiratory conditions (asthma, rare respiratory conditions (cystic fibrosis, bronchiectasis, or alveolitis), chronic obstructive pulmonary disease (COPD), pulmonary hypertension or pulmonary fibrosis); d) cancer (blood cancer, chemotherapy, lung or oral cancer, marrow transplant, radiotherapy); e) neurological conditions (cerebral palsy, Parkinson's disease, rare neurological conditions (motor neurone disease, multiple sclerosis, myasthenia, Huntington's chorea), epilepsy, dementia, learning disability, severe mental illness); and f) other conditions (liver cirrhosis, osteoporotic fracture, rheumatoid arthritis or systemic lupus erythematosus, sickle cell disease, venous thromboembolism, solid organ transplant, renal failure (chronic kidney disease stage 3-5 with or without dialysis or transplant).[61] Body mass index (BMI) will also be considered.[61]

History of healthcare utilisation: Number of GP consultations and hospital admissions in the six months before the start of the study cohort (December 1, 2020) will also be measured as a proxy of severity of pre-existing medical conditions.

History of non-COVID-19 vaccination: Receipt of influenza or pneumococcal vaccination during the 2020-2021 season (1 September to 30 November 2020) available in a binary format (yes or no) will be included which could be predictive of COVID-19 vaccination.

History of prescribed medications: Based on the QCOVID algorithm,[61] we will measure number ($\geq$4) of prescriptions from general practices for oral steroids, long acting β agonists or leukotrienes, immunosuppressants in previous six months prior to the start of the study cohort (1 December 2020).

For peer review only - http://bmjopen.bmj.com/site/about/guidelines.xhtml

History of COVID-19 infection: Any suspected or confirmed COVID-19 infections in the previous last six months (prior to 1 December 2020) will be included.

Statistical analysis

We will report summary statistics of baseline characteristics for the individuals in the study as of 1 December 2020. These will be also stratified by vaccine status and respective study outcome of interest. Relevant measures of tendency (e.g., mean, median, proportion) and variability (e.g., standard deviation, inter-quartile range) will be calculated. Relative estimates (e.g., Odds Ratio (OR) and Rate Ratio (RR)) and their respective 95% Confidence Intervals (CI) for risk of outcomes of interest will also be included for different population strata. Missing data will be reported as percentages of total, imputation will be considered if possible, and sensitivity analysis will be carried out to examine if the nature of the missing data mechanism affects the study findings. All statistical hypotheses tests will be two-tailed with significance level set at 5% for all outcomes of interest. Statistical computing language R will be used to conduct all planned statistical analyses.

Individual-level analyses

Vaccine uptake

Overall proportion of individuals that receive the vaccine, stratified by socio-demographic, medical and other characteristics will be reported. We will also consider reporting uptake levels within certain subgroups of the population where possible (e.g., ethnic minorities and health care and other frontline workers, care home residents and pregnant women). We will also seek to examine patterns related to the number of eligible individuals that were offered vaccine but refused to be vaccinated if data are available. Univariable and multivariable logistic regression models will be carried out to estimate the coefficient of each predictor variable in the model and their 95% CIs, as well as the OR for vaccine uptake.

Vaccine effectiveness

Laboratory confirmed outcomes

Vaccine status will be compared between cases (patients with a positive test for COVID-19) and controls (patients with a negative test for COVID-19) using a TND case-control study.[22] The main advantage of this design compared to traditional case-control studies is that it minimises confounding factors from health care-seeking behaviour, which means both cases and controls have similar likelihood of seeking healthcare when having symptoms indicative of COVID-19 illness.[62] VE is estimated based on the odds ratio (OR) using the formula VE=1-OR. OR is defined as the probability of cases being vaccinated divided by the probability of controls being vaccinated. A generalised additive logistic model will be used to estimate the OR and VE. Stratified VE estimates by vaccine type, dose, dosing schedules, COVID-19 strain, age group and underlying medical condition will also be considered.

The analyses in English databases will be carried out in collaboration with PHE, following their guidance, as the RSC is a major data and sampling source for PHE.[63] It will be important to avoid the confusion that might arise from different approaches to analysing RSC

For peer review only - http://bmjopen.bmj.com/site/about/guidelines.xhtml

data, and to benefit from shared expertise in monitoring VE.[64,65] We have a shared protocol for this season's analysis.[66] In Scotland, all planned analyses will also be carried out in collaboration with PHS. In Wales, analyses will be carried out in collaboration with PHW, who are involved with other colleagues in Wales in the national rollout and evaluation of the vaccine deployment, and are unique placed to provide expertise, and are key contributors as part of the existing ConCOV project in Wales.[24]

Clinical outcomes and deaths

A prospective cohort study will be used to estimate VE against clinical outcomes and deaths in vaccinated and unvaccinated individuals respectively. Time-dependent Cox models will provide the adjusted rate ratios for these outcomes. VE will be calculated according to VE=1-RR. VE estimates will be adjusted for potential confounders and effect modifiers including age, sex, underlying medical condition, SES, history of healthcare utilisation, medication and non-COVID-19 vaccination. Other potential confounders and effect modifiers may also be explored.

Vaccine safety

The risk of any vaccine-related adverse events will be assessed using a SCCS study design.[22] This study design tests whether the risk of an adverse event is higher at post-vaccination period compared to other periods that are temporarily unrelated to vaccine administration.[67] The main advantage of this case series method over other methods of analysis is that it only includes individuals who have been vaccinated. As a result, adequate statistical power can often be obtained with relatively small sample sizes.

In addition, all confounders (e.g., sex, genetics, SES, location, underlying condition) that do not vary with time over the observation period are explicitly controlled for.[67] The number of adverse events in a pre-defined risk interval will be compared to pre-defined control intervals. Risk interval refers to post-vaccine administration period over the observation period of the study and control intervals refer to pre- and post-vaccine administration over the study's observation period. Risk and control intervals will also be determined in relation to vaccine dose administration (e.g., between first and second doses of the vaccines).

The exact duration (in days) of the risk and control intervals will be determined for each AEFI outcome based on severity level (mild-to-moderate, severe and typical onset) and vaccine type separately (see Supplementary Material, Appendix 3). We will use the Benjamini-Hochberg procedure to control the False Discovery rate of testing a large number of hypotheses related to each prespecified adverse events of interest.[68] Sub-group analyses by vaccine type, dose and dosing schedules will also be considered.

It is possible that sample selection bias could be induced in the SCCS if inclusion in the study is related nontrivially to the adverse outcome of interest. This may be particularly true for severe adverse events. For example, if an individual has a cardiac arrest or death then they are less likely to be vaccinated and thus less likely to be included in the study. We will therefore

For peer review only - http://bmjopen.bmj.com/site/about/guidelines.xhtml

carry out a retrospective cohort or an event-dependent exposure version of SCCS study for severe outcomes.

Pooled analyses

In our study, we will initially provide estimates on vaccine uptake, effectiveness, and safety for each UK nation. We will also provide pooled estimates across the UK nations. A generic inverse variance method for meta-analysis will be used. Heterogeneity of our pooled estimates will be assessed using the standard x^2 and the I^2 statistic.[69] Forest plots will be used to visualise any statistical heterogeneity in our pooled estimates for the four nations. Individual nation's ORs or RRs and their 95% CIs will be used to estimate the pooled VE estimates. Measured and unmeasured heterogeneity is highly probable across the UK nations. Effect estimates from random-effect models will thus only be considered.

Sample size

We are basing sample size calculations on Scottish testing and vaccination data because it is currently the only UK nation with full national data coverage. Since the start of the pandemic and as of 8 February 2020, a total of 1,602,405 individuals were tested for COVID-19 infection and of those 186,720 (11.7%) tested positive with an approximately 16% (877,513 vaccinated out of 5.4 million Scottish residents) vaccine uptake coverage.[8] This preliminary calculation gives an 80% power to detect at least a VE of 31% against laboratory confirmed COVID-19 infection.

Patient and Public Involvement and Engagement (PPIE)

PPIE members (Antony Chuter, Alex Brownrigg and Jillian Beggs) have been involved since the beginning of this project. Research proposal for the Wales analysis has also been reviewed by members of the public. Their contribution includes defining research questions, interpretation, and dissemination of study findings. As part of the ConCOV project [24] in Wales, PPIE members have been involved in the design and evaluation of findings, as well as presentations made to the independent SAIL consumer panel group made up of lay members.

Ethics, reporting and dissemination

For Scotland, approvals have been obtained by the National Research Ethics Service Committee (REC), South East Scotland 02 (REC number: 12/SS/0201) and the Public Benefit and Privacy Panel for Health and Social Care (reference number: 1920-0279). The data used in this study are available in the SAIL Databank at Swansea University, Swansea, UK. All proposals to use SAIL data are subject to review by an independent Information Governance Review Panel (IGRP). Before any data can be accessed, approval must be given by the IGRP. The IGRP gives careful consideration to each project to ensure proper and appropriate use of SAIL data. When access has been approved, it is gained through a privacy-protecting safe haven and remote access system referred to as the SAIL Gateway. SAIL has established an application process to be followed by anyone who would like to access data via SAIL.[25] Findings will be presented at conferences, published in peer-reviewed journals and to the funders and government COVID-19 advisory bodies as appropriate. Strengthening the Reporting of Observational Studies in Epidemiology (STROBE) and Reporting of studies

For peer review only - http://bmjopen.bmj.com/site/about/guidelines.xhtml

For peer review only

Conducted using Observational Routinely-collected Data (RECORD) (via the COVID-19 extension) checklists will guide our study findings reporting.[70,71]

Contributors

AS conceived this study and commented on the draft protocol. EV wrote the first draft of the protocol with assistance from TS and SK. SdeL, RT, MJ and DMcG contributed about the English sentinel system and commented on the research methods. All authors contributed to the study design. All authors contributed to drafting the protocol. All authors revised the manuscript for important intellectual content. All authors gave final approval of the version to be published.

Acknowledgments

England: Patients and general practices who provide samples and agree to share data with the Oxford-RCGP RSC. EMIS, TPP, In-Practice Systems, Wellbeing software for supporting the RSC data extraction. Public Health England for support and collaboration. This work uses data provided by patients and collected by the NHS as part of their care and support. We would also like to acknowledge all data providers who make anonymised data available for research. We wish to acknowledge the collaborative partnership that enabled acquisition and access to the de-identified data, which led to this output. The collaboration was led by the Swansea University Health Data Research UK team under the direction of the Welsh Government Technical Advisory Cell (TAC) and includes the following groups and organisations: the Secure Anonymised Information Linkage (SAIL) Databank, Administrative Data Research (ADR) Wales, NHS Wales Informatics Service (NWIS), Public Health Wales, NHS Shared Services Partnership and the Welsh Ambulance Service Trust (WAST). All research conducted has been completed under the permission and approval of the SAIL independent Information Governance Review Panel (IGRP) project number 0911.

Funding

This research is part of the Data and Connectivity National Core Study, led by Health Data Research UK in partnership with the Office for National Statistics and funded by UK Research and Innovation [HDRUK2020.146]. EAVE II is funded by the Medical Research Council [MR/R008345/1] and supported by the Scottish Government. This work is supported by BREATHE - The Health Data Research Hub for Respiratory Health [MC_PC_19004]. BREATHE is funded through the UK Research and Innovation Industrial Strategy Challenge Fund and delivered through Health Data Research UK. ConCOV is supported by the Medical Research Council [MR/V028367/1]; Health Data Research UK [HDR-9006] which receives its funding from the UK Medical Research Council, Engineering and Physical Sciences Research Council, Economic and Social Research Council, Department of Health and Social Care (England), Chief Scientist Office of the Scottish Government Health and Social Care Directorates, Health and Social Care Research and Development Division (Welsh Government), Public Health Agency (Northern Ireland), British Heart Foundation (BHF) and the Wellcome Trust; and Administrative Data Research UK which is funded by the Economic and Social Research Council [grant ES/S007393/1].

Competing interests

AS is a member of the Scottish Government Chief Medical Officer's COVID-19 Advisory Group. RAL reports grants from MRC during the conduct of the study. SJS reports grants from Wellcome Trust, during the conduct of the study; grants from National Institute of Healthcare Research HTA, grants from Tommy's Charity and grants from Chief Scientist for Scotland, outside the submitted work. All other authors report no conflicts of interest.

REFERENCES

1. World Health Organization. Coronavirus disease (COVID-19) outbreak. Available at: https://www.who.int/emergencies/diseases/novel-coronavirus-2019

For peer review only - http://bmjopen.bmj.com/site/about/guidelines.xhtml

2. World Health Organization. Draft landscape of COVID-19 candidate vaccines. Available at: https://www.who.int/publications/m/item/draft-landscape-of-covid-19-candidate-vaccines
3. Regulatory Affairs Professionals Society. COVID-19 vaccine tracker. Available at: https://www.raps.org/news-and-articles/news-articles/2020/3/covid-19-vaccine-tracker
4. European Medicines Agency. COVID-19 vaccines: development, evaluation, approval and monitoring. Available at: https://www.ema.europa.eu/en/human-regulatory/overview/public-health-threats/coronavirus-disease-covid-19/treatments-vaccines/covid-19-vaccines-development-evaluation-approval-monitoring
5. National Health Service. Coronavirus (COVID-19) vaccine. Available at: https://www.nhs.uk/conditions/coronavirus-covid-19/coronavirus-vaccination/coronavirus-vaccine/
6. Polack FP, Thomas SJ, Kitchin N, Absalon J, Gurtman A, Lockhart S, et al. Safety and Efficacy of the BNT162b2 mRNA Covid-19 Vaccine. N Engl J Med. 2020 Dec 31;383(27):2603-2615.
7. Voysey M, Clemens SAC, Madhi SA, Weckx LY, Folegatti PM, Aley PK, et al. Safety and efficacy of the ChAdOx1 nCoV-19 vaccine (AZD1222) against SARS-CoV-2: an interim analysis of four randomised controlled trials in Brazil, South Africa, and the UK. Lancet. 2020 Dec 8:S0140-6736(20)32661-1.
8. UK Government. Coronavirus (COVID-19) in the UK. Available at: https://coronavirus.data.gov.uk/details/healthcare#card-people_who_have_received_vaccinations
9. FDA Briefing Document. Moderna COVID-19 Vaccine. Available at: https://www.fda.gov/media/144434/download
10. Moderna Covid vaccine has 94% efficacy, final results confirm. Available at: https://www.theguardian.com/world/2020/nov/30/moderna-covid-vaccine-has-94-efficacy-final-results-confirm
11. Dhanda S, Osborne V, Lynn E, et alPostmarketing studies: can they provide a safety net for COVID-19 vaccines in the UK? BMJ Evidence-Based Medicine Published Online First: 21 October 2020. doi: 10.1136/bmjebm-2020-111507
12. Dhanda S, Osborne V, Lynn E, Shakir S. Postmarketing studies: can they provide a safety net for COVID-19 vaccines in the UK? BMJ Evidence-Based Medicine Published Online First: 21 October 2020. doi: 10.1136/bmjebm-2020-111507
13. Joint Committee on Vaccination and Immunisation: advice on priority groups for COVID-19 vaccination, 30 December 2020. Available at: https://www.gov.uk/government/publications/priority-groups-for-coronavirus-covid-19-vaccination-advice-from-the-jcvi-30-december-2020/joint-committee-on-vaccination-and-immunisation-advice-on-priority-groups-for-covid-19-vaccination-30-december-2020
14. Iacobucci G, Mahase E. Covid-19 vaccination: What's the evidence for extending the dosing interval? BMJ. 2021 Jan 6;372:n18.

For peer review only - http://bmjopen.bmj.com/site/about/guidelines.xhtml

15. Patel MD, Rosenstrom E, Ivy JS et al. The Joint Impact of COVID-19 Vaccination and Non-Pharmaceutical Interventions on Infections, Hospitalizations, and Mortality: An Agent-Based Simulation. medRxiv 2020.12.30.20248888; doi: https://doi.org/10.1101/2020.12.30.20248888
16. Berlin JA, Glasser SC, Ellenberg SS. Adverse event detection in drug development: recommendations and obligations beyond phase 3. Am J Public Health. 2008;98(8):1366-1371.
17. KFF COVID-19 Vaccine Monitor: December 2020. Available at: https://www.kff.org/coronavirus-covid-19/report/kff-covid-19-vaccine-monitor-december-2020/
18. Madewell ZJ, Yang Y, Longini IM, Halloran ME, Dean NE. Household Transmission of SARS-CoV-2: A Systematic Review and Meta-analysis. JAMA Netw Open. 2020;3(12):e2031756.
19. De Serres G, Skowronski DM, Wu XW, Ambrose CS. The test-negative design: validity, accuracy and precision of vaccine efficacy estimates compared to the gold standard of randomised placebo-controlled clinical trials. Euro Surveill. 2013;18(37):pii=20585.
20. Farrington P, Nash J, Miller E. Case series analysis of adverse reactions to vaccine: a comparative evaluation. Am J Epidem 1996;143(11):1165-73.
21. de Lusignan S, Lopez Bernal J, Zambon M, Akinyemi O, Amirthalingam G, Andrews N, et al. Emergence of a Novel Coronavirus (COVID-19): Protocol for Extending Surveillance Used by the Royal College of General Practitioners Research and Surveillance Centre and Public Health England. JMIR Public Health Surveill. 2020;6(2):e18606.
22. Simpson CR, Robertson C, Vasileiou E, McMenamin J, Gunson R, Ritchie LD, et al. Early Pandemic Evaluation and Enhanced Surveillance of COVID-19 (EAVE II): protocol for an observational study using linked Scottish national data. BMJ Open. 2020;10(6):e039097.
23. Swansea University led team secures funding from COVID-19 Rapid Response Call - Population Data Science. Available at: https://popdatasci.swan.ac.uk/swansea-university-led-team-secures-funding-from-covid-19-rapid-response-call/
24. Lyons J, Akbari A, Torabi F, et al. Understanding and responding to COVID-19 in Wales: protocol for a privacy-protecting data platform for enhanced epidemiology and evaluation of interventions. BMJ Open 2020;10:e043010.
25. The Secure Anonymised Information Linkage (SAIL) databank. Available at: https://saildatabank.com/
26. de Lusignan S, Jones N, Dorward J, Byford R, Liyanage H, Briggs J, et al. The Oxford Royal College of General Practitioners Clinical Informatics Digital Hub: Protocol to Develop Extended COVID-19 Surveillance and Trial Platforms. JMIR Public Health Surveill 2020;6(3):e19773.
27. NHS Digital Data Security and Protection Toolkit. Available at: https://www.dsptoolkit.nhs.uk/
28. NHS Digital. Data Security and Protection Toolkit Organisational Search. University of Oxford - Medical Sciences Division – Nuffield Department of Primary Care Health

For peer review only - http://bmjopen.bmj.com/site/about/guidelines.xhtml

Sciences Organisation code: EE133863-MSD-NDPCHS. Available at: https://www.dsptoolkit.nhs.uk/OrganisationSearch/EE133863-MSD-NDPCHS

29. Public Health England. COVID-19 vaccine surveillance strategy. Available at: https://assets.publishing.service.gov.uk/government/uploads/system/uploads/attachment_data/file/951189/COVID-19_vaccine_surveillance_strategy.pdf
30. de Lusignan S. Codes, classifications, terminologies and nomenclatures: definition, development and application in practice. Inform Prim Care. 2005;13(1):65-70.
31. Oxford-Royal College of General Practitioners (RCGP) Research and Surveillance Centre (RSC). Using Oxford-RCGP RSC for observational studies, surveillance themed dataset. Available at: https://orchid.phc.ox.ac.uk/index.php/orchid-data/
32. de Lusignan S, Williams J. To monitor the COVID-19 pandemic we need better quality primary care data. BJGP Open. 2020;4(2):bjgpopen20X101070.
33. Jani BD, Pell JP, McGagh D, Liyanage H, Kelly D, de Lusignan S, et al. Recording COVID-19 consultations: review of symptoms, risk factors, and proposed SNOMED CT terms. BJGP Open. 2020;4(4):bjgpopen20X101125.
34. de Lusignan S, Liyanage H, McGagh D, Jani BD, Bauwens J, Byford R, et al. COVID-19 Surveillance in a Primary Care Sentinel Network: In-Pandemic Development of an Application Ontology. JMIR Public Health Surveill. 2020 Nov 17;6(4):e21434.
35. COVID-19 vaccination programme in Northern Ireland. Available at: https://www.nidirect.gov.uk/articles/covid-19-vaccination-programme-northern-ireland
36. Turas Vaccination Management Tool. Available at: https://learn.nes.nhs.scot/42708/turas-vaccination-management-tool
37. UK Government. COVID-19 testing data: methodology note. Available at: https://www.gov.uk/government/publications/coronavirus-covid-19-testing-data-methodology/covid-19-testing-data-methodology-note
38. NHS Digital. Data Access Request Service (DARS). Available at: https://digital.nhs.uk/services/data-access-request-service-dars/dars-products-and-services
39. de Lusignan S, Dorward J, Correa A, Jones N, Akinyemi O, Amirthalingam G, et al. Risk factors for SARS-CoV-2 among patients in the Oxford Royal College of General Practitioners Research and Surveillance Centre primary care network: a cross-sectional study. Lancet Infect Dis. 2020;20(9):1034-1042.
40. de Lusignan S, Correa A, Smith GE, Yonova I, Pebody R, Ferreira F, et al. RCGP Research and Surveillance Centre: 50 years' surveillance of influenza, infections, and respiratory conditions. Br J Gen Pract. 2017;67(663):440-441.
41. Department of Health, Northern Ireland. Hospital Statistics. Available at: www.health-ni.gov.uk/articles/inpatient-and-day-case-activity
42. Joy M, Hobbs FDR, McGagh D, Akinyemi O, de Lusignan S. Excess mortality from COVID-19 in an English sentinel network population. Lancet Infect Dis. 2020:S1473-3099(20)30632-0.
43. de Lusignan S, Joy M, Oke J, McGagh D, Nicholson B, Sheppard J, et al. Disparities in the excess risk of mortality in the first wave of COVID-19: Cross sectional study of the English sentinel network. J Infect. 2020;81(5):785-792.
44. Joy M, Hobbs FR, Bernal JL, Sherlock J, Amirthalingam G, McGagh D, et al. Excess mortality in the first COVID pandemic peak: cross-sectional analyses of the impact of

For peer review only - http://bmjopen.bmj.com/site/about/guidelines.xhtml

age, sex, ethnicity, household size, and long-term conditions in people of known SARS-CoV-2 status in England. Br J Gen Pract. 2020;70(701):e890-e898.
45. NHS Digital. National Health Application and Infrastructure Services (NHAIS). Available at: https://digital.nhs.uk/services/nhais
46. Liyanage H, Williams J, Byford R, de Lusignan S. Ontology to identify pregnant women in electronic health records: primary care sentinel network database study. BMJ Health Care Inform. 2019;26(1):e100013.
47. Liyanage H, Williams J, Byford R, Pathirannehelage S, de Lusignan S. Near-Real Time Monitoring of Vaccine Uptake of Pregnant Women in a Primary Care Sentinel Network: Ontological Case Definition Across Heterogeneous Data Sources. Stud Health Technol Inform. 2019;264:1855-1856.
48. Business Services Organisation. Northern Ireland Maternity System (NIMATS). Available at: http://www.hscbusiness.hscni.net/services/2512.htm
49. Stock SJ, McAllister D, Vasileiou E, et al. COVID-19 in Pregnancy in Scotland (COPS): protocol for an observational study using linked Scottish national data. BMJ Open 2020;10:e042813.
50. Moderna. Available at: https://www.modernatx.com/covid19vaccine-eua/providers/clinical-trial-data
51. European Medicines Agency. Interim guidance on enhanced safety surveillance for seasonal influenza vaccines in the EU. Available at: https://www.ema.europa.eu/en/documents/scientific-guideline/interim-guidance-enhanced-safety-surveillance-seasonal-influenza-vaccines-eu_en.pdf
52. Uptake and comparative safety of new COVID-19 vaccines by age, sex, region, ethnicity, comorbidities, medication, deprivation, risk level and evidence of prior COVID infection. Available at: https://www.qresearch.org/research/approved-research-programs-and-projects/uptake-and-comparative-safety-of-new-covid-19-vaccines-by-age-sex-region-ethnicity-comorbidities-medication-deprivation-risk-level-and-evidence-of-prior-covid-infection/
53. National Statistics English indices of deprivation 2019. Available at: https://www.gov.uk/government/statistics/english-indices-of-deprivation-2019
54. de Lusignan S, Sherlock J, Ferreira F, O'Brien S, Joy M. Household presentation of acute gastroenteritis in a primary care sentinel network: retrospective database studies. BMC Public Health. 2020;20(1):445.
55. Welsh Index of Multiple Deprivation 2019. Available at: https://gov.wales/welsh-index-multiple-deprivation-full-index-update-ranks-2019
56. Northern Ireland Multiple Deprivation Measure 2017 (NIMDM2017). Available at: https://www.nisra.gov.uk/statistics/deprivation/northern-ireland-multiple-deprivation-measure-2017-nimdm2017
57. Tippu Z, Correa A, Liyanage H, Burleigh D, McGovern A, Van Vlymen J, et al. Ethnicity Recording in Primary Care Computerised Medical Record Systems: An Ontological Approach. J Innov Health Inform. 2017;23(4):920.
58. Office for National Statistics. Dataset: Lower layer Super Output Area population density (National Statistics), 2020. Available at: https://www.ons.gov.uk/peoplepopulationandcommunity/populationandmigration/populationestimates/datasets/lowersuperoutputareapopulationdensity

For peer review only - http://bmjopen.bmj.com/site/about/guidelines.xhtml

59. de Lusignan S, Sherlock J, Akinyemi O, Pebody R, Elliot A, Byford R, et al. Household presentation of influenza and acute respiratory illnesses to a primary care sentinel network: retrospective database studies (2013-2018). BMC Public Health. 2020;20(1):1748.
60. Northern Ireland Statistics & Research agency. Review of the Statistical Classification and Delineation of Settlements. Available at: https://www.nisra.gov.uk/sites/nisra.gov.uk/files/publications/review-of-the-statistical-classification-and-delineation-of-settlements-march-2015%20%281%29.pdf
61. Clift AK, Coupland CAC, Keogh RH, Diaz-Ordaz K, Williamson E, Harrison EM et al. Living risk prediction algorithm (QCOVID) for risk of hospital admission and mortality from coronavirus 19 in adults: national derivation and validation cohort study BMJ 2020; 371:m3731.
62. Fukushima W, Hirota Y. Basic principles of test-negative design in evaluating influenza vaccine effectiveness. Vaccine. 2017;35(36):4796-4800.
63. Public Health England. COVID-19: vaccine surveillance strategy. London, PHE gateway number GW-1763, 2021. Available at: https://www.gov.uk/government/publications/covid-19-vaccine-surveillance-strategy
64. Pebody RG, Whitaker H, Ellis J, Andrews N, Marques DFP, Cottrell S, et al. End of season influenza vaccine effectiveness in primary care in adults and children in the United Kingdom in 2018/19. Vaccine. 2020;38(3):489-497.
65. Pebody R, Djennad A, Ellis J, Andrews N, Marques DFP, Cottrell S, et al. End of season influenza vaccine effectiveness in adults and children in the United Kingdom in 2017/18. Euro Surveill. 2019;24(31):1800488.
66. de Lusignan S, Lopez Bernal J, Byford R, Amirthalingam G, Ferreira F, Akinyemi O, et al. Seasonal influenza surveillance and vaccine effectiveness at a time of co-circulating COVID-19: Oxford-Royal College of General Practitioners (RCGP) Research and Surveillance Centre (RSC) and Public Health England (PHE) protocol for winter 2020/21 JMIR Public Health and Surveillance. 08/12/2020:24341 (forthcoming/in press) DOI: 10.2196/24341 URL: https://preprints.jmir.org/preprint/24341
67. Petersen I, Douglas I, Whitaker H. Self controlled case series methods: an alternative to standard epidemiological study designs. BMJ 2016;354:i4515.
68. Benjamini, Y. and Hochberg, Y. Controlling the False Discovery Rate: A Practical and Powerful Approach to Multiple Testing. J R Stat Soc Series B Stat Methodol 1995;57:289-300.
69. Higgins JP, Thompson SG, Deeks JJ, Altman DG. Measuring inconsistency in meta-analyses. BMJ. 2003;327(7414):557-560.
70. STROBE Statement. Available at: https://www.strobe-statement.org/index.php?id=strobe-home
71. RECORD. Available at: http://www.record-statement.org/

For peer review only - http://bmjopen.bmj.com/site/about/guidelines.xhtml

Data item	England	Northern Ireland	Scotland	Wales
Exposures				
Pfizer-BioNTech vaccine	GP, NIMS	GP, HSC Trusts	GP, TVMT/PHS, SIRS	CVVD (WIS)
Oxford-AstraZeneca vaccine	GP, NIMS	GP, HSC Trusts	GP, TVMT/PHS, SIRS	CVVD (WIS)
Moderna vaccine	GP, NIMS	GP, HSC Trusts	GP, TVMT/PHS, SIRS	CVVD (WIS)
Laboratory confirmed SARS-CoV-2 infection	GP, Pillar 1&2 SGSS, PHE	Pillar 1 dataset from LIS, Pillar 2 dataset from NHS Digital	ECOSS	PATD (Pillar 1, 2, & 3 data from all NHS and private labs), CVLF testing and results data
Outcomes				
COVID-19 related GP consultation	GP	GP	GP	WLGP
COVID-19 related emergency department consultation	GP, ECDS	Admissions and discharge dataset	SMR01	EDDD & EDDS
COVID-19 related hospital admission	GP, SUS	Admissions and discharge dataset	SMR01	PEDW
COVID-19 related ICU admission	GP, CHESS	Admissions and discharge dataset, ICNARC	SICSAG	CDDS, ICCD & ICNC
COVID-19 related death	GP, ONS, SSRS	NHAIS	NRS	ADDE & ADDE (ONS mortality), CDDS & WDSD
Secondary SARS-CoV-2 infection due to household transmission	RCGP RSC household key	Pillar 1 dataset from LIS, Pillar 2 dataset from NHS Digital	ECOSS	PATD (Pillar 1, 2, & 3 data from all NHS and private labs), CVLF testing and results data
Maternity outcomes	GP, MSDS	NIMATS	COPS study	ADBE (ONS births), MIDS & NCCH
Patient characteristics & confounders				
Age	GP	NHAIS	GP	C19_COHORT20
Sex	GP	NHAIS	GP	C19_COHORT20
Socio-economic status	Post code to IMD	NHAIS	GP	C19_COHORT20
Ethnicity	GP, SUS	To be confirmed	Census 2011	National ethnicity spine (made up of 20 EHR data

Table 1. Available UK datasets for each data item of interest

For peer review only - http://bmjopen.bmj.com/site/about/guidelines.xhtml

				sources and the ONS Census 2011)
Underlying medical condition	GP	To be confirmed	GP	GP, WLGP, PEDW, CVSP
Type of settlement (urban/rural)	GP	NHAIS	GP	C19_COHORT20
Type of settlement (e.g., private home, care home or social housing)	GP	NHAIS	GP	C19_COHORT20, CARE
Smoking status	GP	GP	GP	WLGP
Body Mass Index (BMI)	GP	GP	GP	WLGP
Prescribed medications	GP	EPD	GP, PIS, HEPMA	WLGP, WDDS
Other non-COVID-19 vaccines (e.g., influenza, pneumococcal)	GP	GP	GP	WLGP, NCCH
Occupation (e.g., healthcare workers, frontline workers, essential workers)	GP where recorded	To be confirmed	To be confirmed	HWRA, SWAC
History of healthcare utilisation (e.g., GP consultations, hospital admissions)	GP, SUS	GP, Admissions and discharge dataset	GP, SMR01	PEDW, WLGP

Annual District Birth Extract (ONS Births) (ADBE); Annual District Death Extract (ONS Deaths) (ADDE); COVID Vaccine Data (CVVD); COVID-19 Hospitalisation in England Surveillance System (CHESS); COVID-19 in Pregnancy in Scotland (COPS); COVID-19 Second Generation Surveillance System (SGSS); COVID-19 Shielded People list (CVSP); Critical Care DataSet (CDDS); Electronic Communication of Surveillance in Scotland (ECOSS); Electronic Health Record (HER); Electronic Prescription Database (EPD); Emergency Care Data Set (ECDS); Emergency Department Data Daily (EDDD); Emergency Department Dataset (EDDS); General Practice (GP); Health and Social Care (HSC) Trusts; Hospital Electronic Prescribing and Medicines Administration (HEPMA); Hospital Episode Statistics (HES); ICNARC – Intensive Care National Audit & Research Centre (All admissions) (ICNC); ICNARC – Intensive Care National Audit & Research Centre (COVID-19 Only admissions) (ICCD); Index of Multiple Deprivation (IMD); Intensive Care Admission (ICU); Intensive Care National Audit & Research Centre (ICNARC); Laboratory Information System (LIS); Maternal Indicators DataSet (MIDS); Maternity Services Dataset (MSDS); National Community Child Health database (NCCH); National Health Applications and Infrastructure Services (NHAIS); National Health Service (NHS); National Immunisation Management Service (NIMS); National Records of Scotland (NRS); Northern Ireland Maternity System (NIMATS); Office for National Statistics (ONS); Pathology data COVID-19 Daily (PATD); Patient Episode Database for Wales (PEDW); Prescribing Information System (PIS); Public Health England (PHE); Public Health Scotland (PHS); Royal College of General Practitioners Research and Surveillance Centre (RCGP RSC); Scottish Immunisation & Recall System (SIRS); Scottish Intensive Care Society Audit Group (SICSAG); Scottish Morbidity Record 01 (SMR01); Secondary Users Service (SUS); Turas Vaccination Management Tool (TVMT); Wales Immunisation System (WIS); Welsh Demographic Service Dataset (WDSD); Welsh Longitudinal General Practice (WLGP)

For peer review only

For peer review only - http://bmjopen.bmj.com/site/about/guidelines.xhtml

Supplementary Material

Appendix 1: Adverse Events Following Immunisation (AEFI) reported in pre-licensure COVID-19 vaccine trials

Adverse event	Pfizer-BioNTech (BNT162b2)	Oxford-AstraZeneca (AZD1222)	Moderna
Abdominal pain		Uncommon	
Acute peripheral facial paralysis (/palsy)	Rare (37 days after dose 1, 3-48 days after dose 2)		Rare (22-32 days after dose 2)
Anaphylaxis*	Not known		Not known
Arthralgia	Very common	Very common	Very common
Chills	Very common	Very common	Very common
Decreased appetite		Uncommon	
Dizziness		Uncommon	
Facial swelling			Rare (1-2 days post-vaccination in vaccinees with history of injection of dermatological fillers)
Fatigue	Very common	Very common	Very common
Headache	Very common	Very common	Very common
Hyperhidrosis		Uncommon	
Hypersensitivity	Not known		Not known
Influenza-like illness		Common	
Injection site bruising**		Very common	
Injection site erythema		Very common	Common
Injection site induration		Common	
Injection site pain	Very common	Very common	Very common
Injection site pruritus	Uncommon	Very common	Uncommon
Injection site rash			Common
Injection site redness	Common		
Injection site swelling	Very common	Very common	Very common
Injection site tenderness		Very common	
Injection site urticaria			Common
Injection site warmth		Very common	
Insomnia	Uncommon		
Lymphadenopathy***	Uncommon	Uncommon	Very common
Malaise	Uncommon	Very common	
Myalgia	Very common	Very common	Very common
Nausea****	Common	Very common	Very common
Pain in extremity	Uncommon		
Pruritus		Uncommon	
Pyrexia*****	Very common	Very common	Very common
Rash		Uncommon	Common
Vomiting		Common	
* Anaphylaxis reported in post-marketing setting for Moderna vaccine.			

For peer review only

** Injection site bruising includes injections site haematoma
*** Lymphadenopathy captured as axillary lymphadenopathy on the same side as the injection site for Moderna vaccine.
**** A higher frequency of pyrexia observed after dose 2 for the Pfizer BioNTech vaccine.
***** Pyrexia includes feverishness
Note. Very common (≥1/10), common (≥1/100 to <1/10), uncommon (≥1/1,000 to <1/100), rare (≥1/10,000 to <1/1,000), very rare (<1/10,000), not known (cannot be estimated).

Very rare events of neuroinflammatory disorders have been reported following vaccination with the Oxford AstraZeneca vaccine, but a causal relationship has not been established.

Appendix 2: List of AEFI for influenza vaccines

- Anaphylactic reactions
- Arthropathy
- Bell's palsy
- Conjunctivitis
- Coryza
- Cough
- Decreased appetite
- Diarrhoea
- Drowsiness
- Epistaxis
- Facial oedema
- Fatigue
- Fever / pyrexia
- Guillain-Barré syndrome
- Headache
- Hoarseness
- Hypersensitivity reactions
- Irritability
- Local symptoms (i.e. local erythema)
- Malaise
- Muscle aches / myalgia
- Nasal congestion
- Nausea
- Oropharyngeal pain
- Peripheral tremor
- Rash
- Rhinorrhoea
- Seizure / febrile convulsions
- Vomiting
- Wheezing

For peer review only

For peer review only - http://bmjopen.bmj.com/site/about/guidelines.xhtml

Appendix 3: Schematic presentation of the self-controlled case series study design

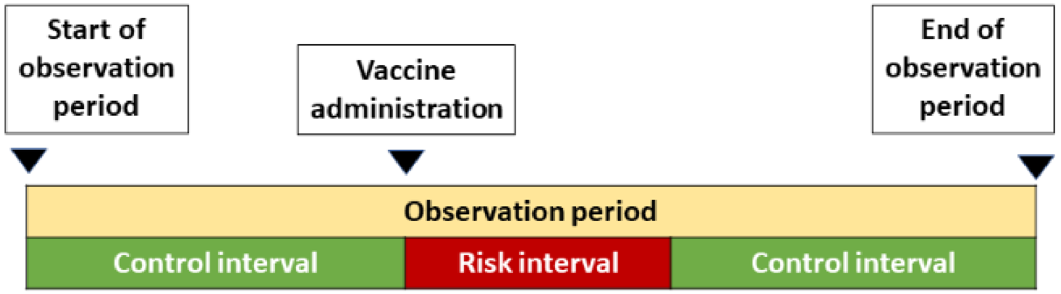

For peer review only

BMJ Open

Investigating the uptake, effectiveness and safety of COVID-19 vaccines: protocol for an observational study using linked UK national data

Journal:	*BMJ Open*
Manuscript ID	bmjopen-2021-050062.R1
Article Type:	Protocol
Date Submitted by the Author:	23-Sep-2021

Complete List of Authors:	Vasileiou, Eleftheria; The University of Edinburgh, Usher Institute<br>Shi, Ting; The University of Edinburgh, Usher Institute<br>Kerr, Steven; The University of Edinburgh, Usher Institute<br>Robertson, Chris; University of Strathclyde, Department of Mathematics and Statistics; Public Health Scotland Glasgow Office<br>Joy, Mark; University of Oxford, Nuffield Department of Primary Care Health Sciences<br>Tsang, Ruby; University of Oxford, Nuffield Department of Primary Care Health Sciences<br>McGagh, Dylan; University of Oxford, Nuffield Department of Primary Care Health Sciences<br>Williams, John; University of Oxford, Nuffield Department of Primary Care Health Sciences<br>Hobbs, Richard; University of Oxford, Nuffield Department of Primary Care Health Sciences<br>de Lusignan, Simon; University of Oxford, Nuffield Department of Primary Care Health Sciences<br>Bradley, Declan; Queen's University Belfast, School of Medicine, Dentistry and Biomedical Sciences<br>OReilly, Dermot; Queen's University Belfast, School of Medicine, Dentistry and Biomedical Sciences<br>Murphy, Siobhan ; Queen's University Belfast, School of Medicine, Dentistry and Biomedical Sciences<br>Chuter, Antony; BREATHE – The Health Data Research Hub for Respiratory Health<br>Beggs, Jillian; BREATHE – The Health Data Research Hub for Respiratory Health<br>Ford, David; Swansea University Medical School, Population Data Science<br>Orton, Chris; Swansea University Medical School, Population Data Science<br>Akbari, Ashley; Swansea University Medical School, Population Data Science<br>Bedston, Stuart; Swansea University Medical School, Population Data Science<br>Davies, Gareth; Swansea University Medical School, Population Data Science<br>Griffiths, Lucy ; Swansea University,<br>Griffiths, Rowena; Swansea University Medical School, Population Data Science

	Lowthian, Emily; Swansea University Medical School, Population Data Science<br>Lyons, Jane; Swansea University Medical School, Population Data Science<br>Lyons, Ronan; Swansea University Medical School, Population Data Science<br>North, Laura; Swansea University Medical School, Population Data Science<br>Perry, Malorie; Public Health Wales, Vaccine Preventable Disease Programme<br>Torabi, Fatemeh; Swansea University Medical School, Population Data Science<br>Pickett, James; Health Data Research UK<br>McMenamin, Jim; Public Health Scotland Glasgow Office<br>McCowan, Colin ; University of St Andrews, School of Medicine<br>Agrawal, Utkarsh; University of St Andrews, School of Medicine<br>Wood, Rachael; Public Health Scotland; The University of Edinburgh, Usher Institute<br>Stock, Sarah; The University of Edinburgh Usher Institute of Population Health Sciences and Informatics, Centre for Medical Informatics; Public Health Scotland,<br>Moore, Emily; Public Health Scotland, Data Driven Innovation<br>Henery, Paul; University of Glasgow, MRC/CSO Social and Public Health Sciences Unit<br>Simpson, Colin; Victoria University of Wellington, Wellington School of Health, Faculty of Health; The University of Edinburgh, Usher Institute<br>Sheikh, Aziz; The University of Edinburgh, Usher Institute
**Primary Subject Heading**:	Epidemiology
Secondary Subject Heading:	Infectious diseases, Respiratory medicine, Public health, Epidemiology, Health informatics
Keywords:	Respiratory infections < THORACIC MEDICINE, Epidemiology < INFECTIOUS DISEASES, Public health < INFECTIOUS DISEASES, COVID-19

SCHOLARONE™
Manuscripts

Investigating the uptake, effectiveness and safety of COVID-19 vaccines: protocol for an observational study using linked UK national data

Dr Eleftheria Vasileiou, Usher Institute, The University of Edinburgh, NINE Edinburgh BioQuarter, Edinburgh, UK, EH16 4UX, eleftheria.vasileiou@ed.ac.uk, +44 07732961139 (corresponding author)

Dr Ting Shi, Usher Institute, The University of Edinburgh, Edinburgh, UK

Dr Steven Kerr, Usher Institute, The University of Edinburgh, Edinburgh, UK

Prof Chris Robertson, Department of Mathematics and Statistics, University of Strathclyde, Glasgow, UK and Public Health Scotland, Glasgow, UK

Dr Mark Joy, Nuffield Department of Primary Care Health Sciences, University of Oxford, Oxford, UK

Dr Ruby Tsang, Nuffield Department of Primary Care Health Sciences, University of Oxford, Oxford, UK

Dylan McGagh, Nuffield Department of Primary Care Health Sciences, University of Oxford, Oxford, UK

John Williams, Nuffield Department of Primary Care Health Sciences, University of Oxford, Oxford, UK

Prof Richard Hobbs, Nuffield Department of Primary Care Health Sciences, University of Oxford, Oxford, UK

Prof Simon de Lusignan, Nuffield Department of Primary Care Health Sciences, University of Oxford, Oxford, UK

Dr Declan Bradley, School of Medicine, Dentistry and Biomedical Sciences, Queen's University Belfast, Belfast, UK

Prof Dermot O'Reilly, School of Medicine, Dentistry and Biomedical Sciences, Queen's University Belfast, Belfast, UK

Dr Siobhan Murphy, School of Medicine, Dentistry and Biomedical Sciences, Queen's University Belfast, Belfast, UK

Antony Chuter, PPIE Lead, BREATHE – The Health Data Research Hub for Respiratory Health, UK

Jillian Beggs, PPIE Lead, BREATHE – The Health Data Research Hub for Respiratory Health, UK

Prof David Ford, Population Data Science, Swansea University Medical School, Swansea, UK

Chris Orton, Population Data Science, Swansea University Medical School, Swansea, UK

For peer review only

Ashley Akbari, Population Data Science, Swansea University Medical School, Swansea, UK

Dr Stuart Bedston, Population Data Science, Swansea University Medical School, UK

Gareth Davies, Population Data Science, Swansea University Medical School, UK

Dr Lucy J Griffiths, Population Data Science, Swansea University Medical School, UK

Rowena Griffiths, Population Data Science, Swansea University Medical School, UK

Emily Lowthian, Population Data Science, Swansea University Medical School, UK

Jane Lyons, Population Data Science, Swansea University Medical School, UK

Prof Ronan A Lyons, Population Data Science, Swansea University Medical School, UK

Laura North, Population Data Science, Swansea University Medical School, UK

Malorie Perry, Vaccine Preventable Disease Programme, Public Health Wales, UK

Fatemeh Torabi, Population Data Science, Swansea University Medical School, UK

Dr James Pickett, Health Data Research UK, London, UK

Dr Jim McMenamin, Public Health Scotland, Glasgow, UK

Prof Colin McCowan, School of Medicine, University of St. Andrews, St Andrews, UK

Dr Utkarsh Agrawal, School of Medicine, University of St. Andrews, St Andrews, UK

Dr Rachael Wood, Usher Institute, The University of Edinburgh, Edinburgh, UK, Public Health Scotland, Edinburgh, UK and Child Life and Health, University of Edinburgh, Edinburgh, UK

Dr Sarah Jane Stock, Tommy's Centre for Maternal and Fetal Health, The University of Edinburgh MRC Centre for Reproductive Health, Edinburgh, UK and Usher Institute, The University of Edinburgh, Edinburgh, UK

Dr Emily Moore, Public Health Scotland, Glasgow, UK

Paul Henery, MRC/CSO Social and Public Health Sciences Unit, University of Glasgow, Glasgow, UK

Prof Colin R Simpson, Wellington School of Health, Faculty of Health, Victoria University of Wellington, Wellington, New Zealand, Usher Institute, The University of Edinburgh, Edinburgh, UK

Prof Aziz Sheikh, Usher Institute, The University of Edinburgh, Edinburgh, UK

Keywords: COVID-19, coronavirus, vaccine, safety, effectiveness, uptake

For peer review

For peer review only - http://bmjopen.bmj.com/site/about/guidelines.xhtml

ABSTRACT

Introduction

The novel coronavirus SARS-CoV-2, which emerged in December 2019 has caused millions of deaths and severe illness worldwide. Numerous vaccines are currently under development of which a few have now been authorised for population level administration by several countries. As of 20 September 2021, over 48 million people have received their first vaccine dose and over 44 million people have received their second vaccine dose across the UK. We aim to assess the uptake rates, effectiveness, and safety of all currently approved COVID-19 vaccines in the UK.

Methods and analysis

We will use prospective cohort study designs to assess vaccine uptake, effectiveness and safety against clinical outcomes and deaths. Test-negative case-control study design will be used to assess vaccine effectiveness (VE) against laboratory confirmed SARS-CoV-2 infection. Self-controlled case series and retrospective cohort study designs will be carried out to assess vaccine safety against mild-to-moderate and severe adverse events, respectively. Individual level pseudonymised data from primary care, secondary care, laboratory test and death records will be linked and analysed in secure research environments in each UK nation. Univariate and multivariate logistic regression models will be carried out to estimate vaccine uptake levels in relation to various population characteristics. VE estimates against laboratory confirmed SARS-CoV-2 infection will be generated using a generalised additive logistic model. Time-dependent Cox models will be used to estimate the vaccine effectiveness against clinical outcomes and deaths. The safety of the vaccines will be assessed using logistic regression models with an offset for the length of the risk period. Where possible, data will be meta-analysed across the UK nations.

Ethics and dissemination

We obtained approvals from the National Research Ethics Service Committee, Southeast Scotland 02 (12/SS/0201), the SAIL independent Information Governance Review Panel (IGRP) project number 0911. Concerning English data, University of Oxford is compliant with the General Data Protection Regulation and the NHS Digital Data Security and Protection Policy. This is an approved study (Integrated Research Application (IRAS) ID 301740, Health Research Authority (HRA) Research Ethics Committee 21/HRA/2786). The Oxford-Royal College of General Practitioners Clinical Informatics Digital Hub (ORCIHD) meets NHS Digital's Data Security and Protection Toolkit requirements. In Northern Ireland the project was approved by the Honest Broker Governance Board (HBGB), project number 0064. Findings will be made available to national policymakers, presented at conferences and published in peer-reviewed journals.

Strengths and limitations of this study

- We will use national data for each UK nation and across the UK general population.

For peer review only - http://bmjopen.bmj.com/site/about/guidelines.xhtml

- Rapid and robust real-time estimates on uptake, effectiveness and safety of COVID-19 vaccines will be provided using data from existing national pandemic platforms in the UK.
- This is an observational study and analyses are therefore potentially susceptible to residual or unmeasured confounders.

For peer review only

For peer review only - http://bmjopen.bmj.com/site/about/guidelines.xhtml

INTRODUCTION

The first human cases infected by the novel coronavirus Severe Acute Respiratory Syndrome Coronavirus 2 (SARS-CoV-2) pathogen were detected in Wuhan, China in December 2019.[1] On 11 March 2020, the World Health Organization (WHO) declared the coronavirus 2019 disease (COVID-19) as a global pandemic, which as of 20 September 2021 has caused more than 228 million infections and four million deaths worldwide.[1] The need for vaccines against this novel virus triggered an emergency response by governments, pharmaceutical companies, and research institutions to develop, licence and manufacture COVID-19 vaccines at scale. Dozens of COVID-19 vaccines are currently under development with some vaccines now having successfully completed their pre-licensure clinical trials and been approved for population vaccine administration.[2,3] The speed with which the world's first vaccines [3] were available for mass administration at the end of 2020 is unprecedented given that typically it takes years for a vaccine to be available for use at a population level.[4]

The Pfizer-BioNTech and Oxford-AstraZeneca were amongst the first vaccines approved by national regulatory authorities such as the United Kingdom's (UK) Medicines and Healthcare products Regulatory Agency (MHRA).[5] Based on pre-licensure clinical trials, the Pfizer-BioNTech vaccine was 95% effective at preventing laboratory-confirmed COVID-19 illness in individuals without evidence of previous infection.[6] The Oxford-AstraZeneca vaccine has also reported significant efficacy of 64% and 70% after one and two doses, respectively against symptomatic disease.[7] As of the 8 January 2021, the UK has also approved a third COVID-19 vaccine manufactured by Moderna which has shown 94% efficacy against confirmed SARS-CoV-2 infection after receipt of second dose.[8] All three vaccines were well tolerated with mild-to-moderate side-effects mostly reported.[6,7,8] In March 2021, the European Medicines Agency (EMA) reported extremely rare but serious side-effects including blood clots and bleeding following administration of the Oxford-AstraZeneca vaccine.[9,10] As a result, the Joint Committee on Vaccination and Immunisation (JCVI) of the UK Government has recommended that healthy adults aged 18-39 years olds should not be offered the Oxford-AstraZeneca vaccine but the Pfizer-BioNTech or Moderna vaccines instead.[11] Rare cases of cardiac inflammation including myocarditis and pericarditis following immunisation with the Pfizer-BioNTech and Moderna vaccines have also been reported [12] and are thus closely monitored by national and international regulatory agencies.

As of 20 September 2021, over 48 million people have received their first vaccine dose and over 44 million people have received their second vaccine dose across the UK [13] based on JCVI's vaccination priority list which targets those most at risk of severe COVID-19 illness (e.g., older adults and people with comorbidities).[14]

Timely post-licensure monitoring of coverage, protection and safety of these newly introduced vaccines is imperative.[15] Specifically, robust observational epidemiological studies are required to measure coverage rates in the population in relation to demographic and other population characteristics, assess effectiveness against infection, severe illness and deaths, and to detect adverse events. These post-marketing observational studies will add additional value to the pre-licensure clinical trials as they can assess real-life effects of the COVID-19 vaccines

For peer review only - http://bmjopen.bmj.com/site/about/guidelines.xhtml

and the impact of the vaccination programme at population levels.[15] All UK-licensed vaccines have demonstrated high efficacy in clinical trials; however, more evidence is needed about the type, level, and duration of protection for different segments of the population. The recommended time period between administration of the first and second doses of the Pfizer-BioNTech [6] and Oxford-AstraZeneca [7] vaccines are 3 and 6 weeks, respectively. The UK government decided to lengthen this gap to up to 12 weeks for both vaccines.[14] This is because of the desire to provide some degree of protection to as many people as possible and the decision was supported by findings from the Oxford-AstraZeneca vaccine trial, which have shown that vaccine efficacy was higher (65%) in the group that was administered the second dose more than six weeks after the first dose, compared to the group given the second dose less than six weeks after the first dose (53%).[7,16] A simulation study has also shown that high vaccine coverage even with less efficacious vaccines (due to partial immunisation) can lead to a greater reduction of SARS-CoV-2 infection levels compared to a lower vaccine coverage with more efficacious vaccines.[17] On the other hand, some experts have expressed their concerns that delay between doses could increase the risk of vaccine-resistant strains emerging due to a partially immunised population.[16] The provision of timely estimates on the protection conferred between doses is thus urgently needed. The assessment of vaccine-induced adverse events also needs to be carried out, particularly for rare adverse events that are usually only detectable in large population studies.[18] The rate of vaccine receipt by demographic, socioeconomic and other epidemiological characteristics also needs to be measured. In the UK, the 'Understanding Society' COVID-19 survey asked 12,035 participants (in November 2020) their likelihood of vaccine uptake and reason for hesitancy.[19] High levels of hesitancy were found in women (21%), younger adults aged 16-24 years olds (27%), those with lower education levels (19%) and in Black (72%) and Pakistani/Bangladeshi (42%) ethnic groups.[19] This is particularly concerning given that ethnic minority groups are some of the subgroups of the population that have been most at risk in this pandemic.[19] The identification of key factors related to vaccine uptake may be useful in efforts to increase uptake and subsequently maximise the impact of the COVID-19 vaccination programme in the UK.

Aim and objectives The aims of this study are to assess the uptake, effectiveness, and safety of the currently licenced COVID-19 vaccines (Pfizer-BioNTech, Oxford-AstraZeneca and Moderna) in the UK using linked healthcare and administrative data. We will also seek to assess any additional licensed vaccines during the course of this study.

Our primary objectives are to: a) measure variation in vaccine uptake in relation to population characteristics; b) assess vaccine effectiveness (VE) against infection, transmission, [20] severe outcomes, and deaths; and c) identify the risk of adverse events following immunisation (AEFIs) in each UK nation. Our secondary objectives are to provide UK-wide pooled estimates of each primary objective.

METHODS

Study design

For peer review only - http://bmjopen.bmj.com/site/about/guidelines.xhtml

A prospective cohort study design will be used to measure variations in vaccine uptake and assess VE against severe illness, deaths, secondary SARS-CoV-2 infection due to household transmission. A test-negative design (TND) case-control study will be carried out to assess VE against laboratory confirmed COVID-19 infection. In the TND, cases and controls will be those with a positive and negative test for COVID-19, respectively.[21] A self-controlled case series (SCCS) study design will be used to assess the risk of AEFI. The SCCS study will be used to determine the relative incidence of adverse events for exposed time periods (periods following vaccine administration) compared to unexposed time periods (pre- or post-vaccination periods unrelated to vaccination) in individuals who present with the outcome of interest.[22] For more severe and event dependent safety outcomes, a retrospective cohort study will be considered.

Overview of linked databases and study population

We will use pseudonymised individual level data routinely collected at primary and secondary care visits, linked with mortality, laboratory, and vaccination data across the UK. Unique national datasets will be developed and hosted within secure research environments in each UK nation with standardised individual-level analyses run across datasets. Pooled estimates across the UK nations will also be calculated. For England, data from the Royal College of General Practitioners Research and Surveillance Centre (RCGP RSC) (approximately 5.4 million people) [23] will be used. We will also access and analyse national coverage data from Northern Ireland (approximately 1.9 million people). For Scotland, data will be derived from the Early Pandemic Evaluation and Enhanced Surveillance of COVID-19 (EAVE II) (approximately 5.4 million people).[24] For Wales, data from the Controlling COVID19 through enhanced population surveillance and intervention (ConCOV) (approximately 3.2 million people) [25,26] and the Secure Anonymised Information Linkage (SAIL) Databank [27] will be used. See Table 1 for details on data sources from each UK nation.

England

English primary care data will be held in the Oxford-Royal College of General Practitioners Clinical Informatics Digital Hub (ORCHID).[28] Data will be pseudonymised as close to source as possible using an NHS Digital Data Security and Protection (DSP) approved method.[29,30] NHS Digital will link additional data to these pseudonyms.

Northern Ireland

For the Northern Irish data, the Honest Broker Service will carry out the data linkage and offer a secure environment for researchers to access and analyse the pseudonymised individual level data. The Health Care Number (HCN) will be used to link individuals' data though replaced with an anonymous study ID in the analysis dataset.

Scotland

For the Scottish data, Public Health Scotland (PHS) will carry out the data linkage and offer a secure environment for researchers to access and analyse the pseudonymised individual level data.[24] The Community Health Index (CHI) number (a unique identifier for each resident receiving healthcare) will be used to link individuals' data which will be replaced by a study ID.

For peer review only

Wales
Withing SAIL and in collaboration with the ConCOV project,[25,26] data will be linked and anonymised from NHS sources via a mature split-file system. All identifiable data will stay within the NHS, and will be linked to the Welsh Demographic Service, pseudonymised, and encrypted within NHS Wales by Digital Health and Care Wales (DHCW), with pseudonymised demographics supplied to SAIL at Swansea University,[27] where the non-identifiable clinical data are held. SAIL will link the demographic data to relevant clinical data, and then further encrypt the linked data before presenting to research teams within a secure virtual desktop.

Exposure (vaccination) data
England
In England, data on vaccination will derive from GPs and the National Immunisation Management Service (NIMS).[31] NIMS is the System of Record for the NHS COVID-19 vaccination programme in England developed by NHS Digital.[31] NIMS will collect any demographic, GP and employee (for NHS) data to identify groups of the population eligible for vaccination. Data collected from NIMS will also feed back into GP systems so that an individual's electronic health record is updated regarding to their vaccination history.[31] Vaccination data in GP records will be recorded using the Systematised Nomenclature of Medicine (SNOMED) Clinical Terms (CT).[32 RCGP RSC now uses SNOMED CT for all its key variables including vaccine data.[33] A key part of these curated variables are those for COVID-19; there have been three iterations of these and we have carefully curated case definitions.[34-36]

Northern Ireland
In Northern Ireland, GP practices and Trust vaccination sites are currently delivering the COVID-19 vaccination programme. Vaccination data from GP practices and Trust vaccination sites are stored in a central Vaccination Management System (VMS) and made available via the Honest Broker Service (HBS) secure research environment.[37]

Scotland
In Scotland, General Practitioners (GPs) usually facilitate vaccination programmes and record any data related to vaccine administration. GPs use Read codes to code and record relevant information arising from a patient consultation.[24] Additional data on vaccination will also be available via the Turas Vaccination Management Tool (TVMT), which is a web-based tool that enables frontline vaccinators to capture and create real-time patient vaccination records. PHS is currently collating vaccination data from Turas.[38] Scheduled vaccinations for children may also be recorded in the Scottish Immunisation Recall System (SIRS) database. Data on vaccinations administered at schools and not in GPs may thus derive from SIRS.[24]

Wales
In Wales, the vaccine programme is administered and recorded nationally in the all Wales Immunisation System (WIS) and is available in SAIL through the COVID Vaccine Data (CVVD). This is a separate independent system and data source to the GP data, which is also available in SAIL with vaccination recorded using Read codes.[27]

For peer review only - http://bmjopen.bmj.com/site/about/guidelines.xhtml

Outcome data

- *Laboratory confirmed outcomes*

England

For England, most community testing (called Pillar 2) is in the GP record, though for this study we will additionally link to resources held by NHS Digital this is the Second Generation Surveillance System (SGSS), this also included hospital tests.[39]

Northern Ireland

For Northern Ireland, the Pillar 1 dataset is extracted from the Laboratory Information Systems in Northern Ireland hospitals on a daily basis into a central repository in the Health and Social Care (HSC) Regional Data Warehouse, which is maintained by the HSC Business Services Organisation.[39] It contains details of COVID-19 antigen tests carried out in each of the hospital laboratories, including those processed on behalf of primary care, social care and community settings. Pillar 2 data are processed by NHS Digital and extracts for NI residents are sent to the NI HSC Regional Data Warehouse.[39]

Scotland

For Scotland, laboratory results from Scottish diagnostic and reference laboratories are captured by the Electronic Communication of Surveillance in Scotland (ECOSS) database, which can be used for surveillance and research purposes.[24] Laboratory results from NHS and community (Lighthouse laboratory) testing centres will also be available through ECOSS.

Wales

For Wales, national coverage of Pillar 1, 2 and 3 data from all NHS and private laboratories will be available, as well as national lateral flow testing data.[39]

We will also pursue to access genome sequencing data in a proportion of laboratory tests positive for SARS-CoV-2 available from national sequencing centres where possible.

- *Clinical outcomes*

Data on primary care consultations for COVID-19 illness will be accessed via GP electronic health records in each UK nation.

England

In England, we will link to secondary care data through collections held by NHS Digital. Hospital data are held in two forms: Hospital Episode Statistics (HES), which are the long-term validated record; and Secondary Uses Services (SUS) which is an extract of contemporary operational data which after validation will become HES. We plan to use HES and also have an extract of intensive care data. We will access these data through NHS Digital's Data Access Request Service (DARS).[40] We also have SARS-CoV-2 virology data that were collected through the sentinel surveillance system.[41] The English primary care sentinel system within RCGP RSC has a strong working relationship with Public Health England (PHE), with whom we have worked closely for over half a century.[42]

For peer review only - http://bmjopen.bmj.com/site/about/guidelines.xhtml

Northern Ireland
In Northern Ireland, the Admissions and Discharge dataset will be used to collate data relating to admitted patient care delivered by HSC hospitals in Northern Ireland, generated by the patient administration systems within each hospital. These data are held centrally by the HSC Regional Data Warehouse.[43]

Scotland
In Scotland, data on patients receiving care in general or acute hospitals are recorded in the Scottish Morbidity Record 01 (SMR01).[24] The International Statistical Classification of Diseases and Related Health Problems, Tenth Revision (ICD-10) codes are used to index any diagnoses recorded in the patient's medical notes by a clinician.[24] Consistent and high level (>90%) of data accuracy have been shown in recent data quality reports for the SMR01 database.[20] Data on adult patients admitted to general intensive care units will derive from the Scottish Intensive Care Society Audit Group (SICSAG) database.[24]

Wales
In Wales, data on all interactions with secondary care including Accident and Emergency events (Emergency Department Dataset – EDDS), inpatient hospital admissions (Patient Episode Database for Wales - PEDW), intensive care (Critical Care DataSet – CDDS and Intensive Care National Audit & Research Centre - ICNARC) and outpatient appointments and activity (Outpatient Database for Wales – OPDW) are all available within SAIL Databank.[27] There are also a collection of specialised services and condition specific secondary care such as cancer which are available within SAIL.[27]

- *Deaths*

England
In England, NHS Digital's Personal Demographic Service flags the date of death in the GP record. We have previously used these data of death to report excess mortality, both overall [44,45] and in people with known COVID-19 status.[46] We will augment these data with the certificated cause of death provided by the Office for National Statistics (ONS), which will be linked for us at individual pseudonymised patient level by NHS Digital.

Northern Ireland
In Northern Ireland, the National Health Applications and Infrastructure Services (NHAIS) will be used for mortality data.[47] NHAIS receives regular updates from GRO (General Register Office) on fact and cause of death. ICD-10 codes on deaths records derive from diagnoses recorded by the certifying doctor on the death certificate. [47]

Scotland
In Scotland, the death registry within National Records of Scotland (NRS) records information included in the death certificates.[24] ICD-10 codes on deaths records derive from diagnoses recorded by the certifying doctor on the death certificate.[24]

Wales

For peer review only - http://bmjopen.bmj.com/site/about/guidelines.xhtml

In Wales, multiple sources of mortality data including information from the National Population Spine (Welsh Demographic Service – WDSD), ONS death data (ADDE and ADDD) and a national NHS master patient index record (Consolidated Death Data Source – CDDS) will be accessed to retrieve complete, harmonised details on cause and associated mortality details.[27]

- *Pregnancy and neonatal outcomes*

Capturing vaccine exposure in pregnancy is important.[48] As no trial to date has included pregnant women this type of study is the only opportunity to explore safety and effectiveness in pregnant women and their babies.

England

In England, we will use a customised "sliding window" to capture pregnancy data.[49] Specifically, an algorithm that accurately inferred pregnancies will be used by adopting an ontological approach for case finding.[49] The ontological approach will thus be used to identify pregnancies and associated complications using a systematic approach to derive this information from routinely collected administrative health data which will be available via the RCGP RSC.[49]

Northern Ireland

In Northern Ireland, data will be accessed via the Northern Ireland Maternity System (NIMATS).[50]

Scotland

In Scotland, pregnancy and neonatal outcomes in Scottish participants will be identified through the COVID-19 in Pregnancy in Scotland (COPS) study.[51]

Wales

In Wales, pregnancy and neonatal outcomes will be identified from linked Annual District Birth Extract (ADBE), National Community Child Health (NCCH) and Maternity Indicators DataSet (MIDS). Data sources for Wales will be sought via the SAIL Databank.[27]

Exposure definitions

Data on the currently licenced COVID-19 vaccines, including Pfizer-BioNTech, Oxford-AstraZeneca and Moderna, will be derived from GPs, National Immunisation Management Service (NIMS), TVMT/PHS, WIS and HSC Trusts databases. For the first vaccine dose (partial vaccination), an individual will be defined as exposed or vaccinated from day 14 after receiving the first dose between the period of 8 December 2020 and until the end of follow up. For the second vaccine dose (full vaccination), an individual will be defined as exposed or vaccinated from day 14 after receiving the second dose during the study period. Exposed or vaccinated groups will be stratified by the following time intervals: a) 0-13 days after dose 1; b) 14-20 days after dose 1; c) 21-27 days after dose 1; d) 28-34 days after dose 1; e) 35-41 days after dose 1; f) ≥42 days after dose 1; g) 0-13 days after dose 2 and h) ≥14 days after dose 2.

Controls or unvaccinated will be defined as those who have not yet received a COVID-19 vaccine or have only received one vaccine dose. Controls who become vaccinated with any

For peer review only - http://bmjopen.bmj.com/site/about/guidelines.xhtml

vaccine (i.e., including one dose of Moderna) or receive a second vaccine will then be assigned within the exposure group. As a result, follow-up of the exposure period will be censored for both the vaccinated and control recipient if the control meets the criteria to be classified as exposed (receiving a first dose when compared with the unvaccinated group and receiving a second dose when compared with the partially/one dose vaccinated group). Maximum follow-up period will correspond to the latest event date depending on the outcome of interest. Similar vaccinated and unvaccinated groups and periods will be determined for the Moderna vaccine.[52]

Outcome definitions

Adverse events following immunisation (AEFI): Adverse events to be monitored are derived from the safety results of the pre-licensure vaccine clinical trials, common side-effects related to influenza vaccines and an unpublished study protocol of an ongoing observational study.[6,7,53,54] These include use of health services such as GP or out-of-hours GP consultation, accident and emergency (A&E) department attendance, inpatient hospital admission and admission to intensive care units (ICU) for suspected adverse events. Safety of vaccines in pregnant women will also be considered once vaccines are widely administered in this group of the population. AEFI by specific vaccine type will also be considered. A full list of candidate AEFI is available in in the Supplementary Material, Appendix 1-2.

Vaccine effectiveness outcomes: Protective effects of the vaccines will be assessed against the following outcomes: a) RT-PCR laboratory confirmed COVID-19 infection; b) GP consultations related to suspected or confirmed COVID-19 illness; c) A&E attendance related to suspected or confirmed COVID-19 illness at presentation; d) hospital admissions related to confirmed COVID-19 illness; e) ICU admissions related to confirmed COVID-19 illness; and f) deaths related to suspected or confirmed COVID-19 illness. We will also explore the effects of vaccines on secondary SARS-CoV-2 infection due to household transmission. VE against maternal and neonatal COVID-19 related outcomes will be explored once the vaccines are more widely available to pregnant women.

A COVID-19 hospital or ICU admission will be defined based on either a RT-PCR confirmed positive test for SARS-CoV-2 in the 28 days prior to admission or based on an ICD-10 code for COVID-19 (U07.1 or U07.2) in any diagnostic position. A COVID-19 death will be defined as COVID-19 as the underling ICD-10 cause of death recorded on the death certificate, or death from any cause within 28 days of a positive RT-PCR test for SARS-CoV-2.

Population characteristics, confounding factors, and effect modifiers

A number of key characteristics that could explain variation in vaccine uptake will be considered. In addition, these characteristics could confound our planned analyses. We will determine and include these characteristics or potential confounders at the baseline of our study's cohort which include (see Table 1 for details on data sources for each UK nation):

For peer review only - http://bmjopen.bmj.com/site/about/guidelines.xhtml

Socio-demographics: Sex at birth will be included in a binary format (females and males). Age will be included in bands that will be determined based on available vaccination data. Socio-economic status will be assessed by the following national versions of area level deprivation indices: a) Index of Multiple Deprivation (IMD), 2019 version for England [55,56]; b) the Scottish Index of Multiple Deprivation (SIMD),[24]; c) the Welsh Index of Multiple Deprivation (WIMD) [57] and d) the Northern Ireland Multiple Deprivation Measure (NIMDM).[58] For England, we will maximise the capture of ethnicity data through the use of a customised ontology.[59] Ethnicity data for the other UK nations will also be included if available.

Geographic: In England, the RSC will use the ONS data on population density and classify households as rural, urban (town and city), or conurbation.[60] The RSC also has a unique household key, so we can report household incidence of respiratory and other infectious conditions.[61] In Scotland, settlement type will be included using the urban/rural 6-fold classification (UR6).[24] In Wales, urban and rural household classification that is based on the Lower-layer Super Output Area (LSOA) of the person's residence information will be used.[26] In Northern Ireland, NISRA's Statistical Classification and Delineation of Settlements will be used to determine settlement type.[62] Type of residence will also be considered such as private residence, care home and social/council housing for each UK nation if data are available.

Smoking status: Smoking status will be included through four categories: smoker, ex-smoker, non-smoker and 'not recorded'. Smoking status will not be determined for Northern Ireland due to no access to primary care data.

At-risk underlying medical conditions: Based on the QCOVID algorithm [63] we will consider the following conditions: a) cardiovascular conditions (atrial fibrillation, heart failure, stroke, peripheral vascular disease, coronary heart disease, congenital heart disease); b) diabetes (type 1 and type 2); c) respiratory conditions (asthma, rare respiratory conditions (cystic fibrosis, bronchiectasis, or alveolitis), chronic obstructive pulmonary disease (COPD), pulmonary hypertension or pulmonary fibrosis); d) cancer (blood cancer, chemotherapy, lung or oral cancer, marrow transplant, radiotherapy); e) neurological conditions (cerebral palsy, Parkinson's disease, rare neurological conditions (motor neurone disease, multiple sclerosis, myasthenia, Huntington's chorea), epilepsy, dementia, learning disability, severe mental illness); and f) other conditions (liver cirrhosis, osteoporotic fracture, rheumatoid arthritis or systemic lupus erythematosus, sickle cell disease, venous thromboembolism, solid organ transplant, renal failure (chronic kidney disease stage 3-5 with or without dialysis or transplant).[63] Body mass index (BMI) will also be considered.[63]

History of healthcare utilisation: Number of GP consultations and hospital admissions in the six months before the start of the study cohort (December 1, 2020) will also be measured as a proxy of severity of pre-existing medical conditions.

For peer review only - http://bmjopen.bmj.com/site/about/guidelines.xhtml

History of non-COVID-19 vaccination: Receipt of influenza or pneumococcal vaccination during the 2020-2021 season (1 September to 30 November 2020) available in a binary format (yes or no) will be included which could be predictive of COVID-19 vaccination.

History of prescribed medications: Based on the QCOVID algorithm,[63] we will measure number ($\geq$4) of prescriptions from general practices for oral steroids, long acting β agonists or leukotrienes, immunosuppressants in previous six months prior to the start of the study cohort (1 December 2020). Prior or concomitant usage of the novel oral anticoagulants (NOACs), warfarin and heparin will also be measured.

History of COVID-19 infection: Any suspected or confirmed COVID-19 infections in the previous last six months (prior to 1 December 2020) will be included.

Statistical analysis

We will report summary statistics of baseline characteristics for the individuals in the study as of 1 December 2020. These will be also stratified by vaccine status and respective study outcome of interest. Relevant measures of tendency (e.g., mean, median, proportion) and variability (e.g., standard deviation, inter-quartile range) will be calculated. Relative estimates (e.g., Odds Ratio (OR) and Rate Ratio (RR)) and their respective 95% Confidence Intervals (CI) for risk of outcomes of interest will also be included for different population strata. Missing data will be reported as percentages of total, imputation will be considered if possible, and sensitivity analysis will be carried out to examine if the nature of the missing data mechanism affects the study findings. All statistical hypotheses tests will be two-tailed with significance level set at 5% for all outcomes of interest. Statistical computing language R will be used to conduct all planned statistical analyses.

Individual patient-level analyses

Vaccine uptake

Prospective cohort study for vaccine uptake

Overall proportion of individuals that receive the vaccine, stratified by socio-demographic, medical and other characteristics will be reported using a prospective cohort study. We will also consider reporting uptake levels within certain subgroups of the population where possible (e.g., ethnic minorities and health care and other frontline workers, care home residents and pregnant women). We will also seek to examine patterns related to the number of eligible individuals that were offered vaccine but refused to be vaccinated if data are available. Univariable and multivariable logistic regression models will be carried out to estimate the coefficient of each predictor variable in the model and their 95% CIs, as well as the OR for vaccine uptake.

Vaccine effectiveness

Test negative design case-control study for laboratory confirmed outcomes

Vaccine status will be compared between cases (patients with a positive test for COVID-19) and controls (patients with a negative test for COVID-19) using a TND case-control study.[24]

For peer review only - http://bmjopen.bmj.com/site/about/guidelines.xhtml

The main advantage of the TND studies compared to traditional case-control studies is that it minimises confounding factors from health care-seeking behaviour, which means both cases and controls have similar likelihood of seeking healthcare when having symptoms indicative of COVID-19 illness.[64] Selection bias can still arise if study participants are not recruited based on pre-defined criteria (e.g., signs/symptoms indicative to COVID-19 illness) but based on clinician-ordered test.[64] In this scenario, clinicians may be more likely to carried out a test on patients that are more likely to have COVID-19 illness (outcome) or not being vaccinated.[64] This will result to biased sampling (non-representativeness) of the study participants from the source population which could lead to overestimation of the VE estimates.[64] VE is estimated based on the odds ratio (OR) using the formula VE=1-OR. OR is defined as the odds of a SARS-CoV-2 infection among the vaccinated group divided by the odds of a SARS-CoV-2 infection among the unvaccinated group.. A generalised additive logistic model will be used to estimate the OR and VE. Stratified VE estimates by vaccine type, dose, dosing schedules, COVID-19 strain, age group and underlying medical condition will also be considered.

The analyses in English databases will be carried out in collaboration with PHE, following their guidance, as the RSC is a major data and sampling source for PHE.[65] It will be important to avoid the confusion that might arise from different approaches to analysing RSC data, and to benefit from shared expertise in monitoring VE.[66,67] We have a shared protocol for this season's analysis.[68] Likewise, in Northern Ireland, we will work closely with the Public Health Agency. In Scotland, all planned analyses will also be carried out in collaboration with PHS. In Wales, analyses will be carried out in collaboration with PHW, who are involved with other colleagues in Wales in the national rollout and evaluation of the vaccine deployment, and are unique placed to provide expertise, and are key contributors as part of the existing ConCOV project in Wales.[26]

Prospective cohort study for clinical outcomes and deaths

A prospective cohort study will be used to estimate VE against clinical outcomes and deaths in vaccinated and unvaccinated individuals respectively. Time-dependent Cox models will provide the adjusted rate ratios for these outcomes. VE will be calculated according to VE=1-RR. VE estimates will be adjusted for potential confounders and effect modifiers including age, sex, underlying medical condition, SES, history of healthcare utilisation, medication and non-COVID-19 vaccination. Other potential confounders and effect modifiers may also be explored. Recall and misclassification bias will be minimised in our planned prospective cohort studies as we will use data from national linked datasets which allow rapid analysis of vaccination and clinical outcomes data derived from electronic health records. Nevertheless, unmeasured confounding can still influence the VE estimates (given the observational nature of these study designs) despite attempts to provide VE adjusted for potential confounders as mentioned above.

Additional sensitivity or post-hoc analyses such as using different time intervals following administration of the vaccine to define exposure will also be explored for all study outcomes related to vaccine effectiveness.

For peer review only - http://bmjopen.bmj.com/site/about/guidelines.xhtml

Vaccine safety

Self-controlled case series and retrospective cohort studies for adverse events

The risk of any vaccine-related adverse events will be assessed using a SCCS study design.[24] This study design tests whether the risk of an adverse event is higher at post-vaccination period compared to other periods that are temporarily unrelated to vaccine administration.[69] The main advantage of this case series method over other methods of analysis is that it only includes individuals who have been vaccinated. As a result, adequate statistical power can often be obtained with relatively small sample sizes.

In addition, all confounders (e.g., sex, genetics, SES, location, underlying condition) that do not vary with time over the observation period are implicitly controlled for.[69] The number of adverse events in a pre-defined risk interval will be compared to pre-defined control intervals. Risk interval refers to post-vaccine administration period over the observation period of the study and control intervals refer to pre- and post-vaccine administration over the study's observation period. Risk and control intervals will also be determined in relation to vaccine dose administration (e.g., between first and second doses of the vaccines). A clearance or wash-out interval between the risk and control intervals will also be applied.

The exact duration (in days) of the risk and control intervals will be determined for each AEFI outcome based on severity level (mild-to-moderate, severe and typical onset) and vaccine type separately (see Supplementary Material, Appendix 3). We will use the Benjamini-Hochberg procedure to control the False Discovery rate of testing a large number of hypotheses related to each prespecified adverse events of interest.[70] Sub-group analyses by vaccine type, dose and dosing schedules will also be considered.

It is possible that sample selection bias could be induced in the SCCS if inclusion in the study is related nontrivially to the adverse outcome of interest. This may be particularly true for severe adverse events. For example, if an individual has a cardiac arrest then they are less likely to be vaccinated and thus less likely to be included in the study. We will therefore carry out a retrospective cohort or an event-dependent exposure version of SCCS study for severe outcomes.

A sensitivity analysis by previous history of SARS-CoV-2 infection will also be considered. We will explore if previous SARS-CoV-2 infection is associated with any AEs observed following a COVID-19 vaccination.

Pooled analyses

In our study, we will initially provide estimates on vaccine uptake, effectiveness, and safety for each UK nation. We will also provide pooled estimates across the UK nations. A generic inverse variance method for meta-analysis will be used. Heterogeneity of our pooled estimates will be assessed using the standard X^2 and the I^2 statistic.[71] Forest plots will be used to visualise any statistical heterogeneity in our pooled estimates for the four nations. Individual nation's ORs or RRs and their 95% CIs will be used to estimate the pooled VE estimates.

For peer review only - http://bmjopen.bmj.com/site/about/guidelines.xhtml

Measured and unmeasured heterogeneity is highly probable across the UK nations. Effect estimates from random-effect models will thus only be considered.

Sample size

We are basing sample size calculations on Scottish testing and vaccination data because it is currently the only UK nation with full national data coverage at the time of writing. Based on previous work,[72] we estimated a VE of 0.89 against COVID-19 hospitalisation at 28-34 days post vaccination, with a standard deviation of 0.06. Assuming our VE estimates are asymptotically normally distributed, this gives almost 100% power to detect a VE of over 0.5.

The number of COVID-10 vaccines doses required to detect a relative risk of 5.0 is at least 10,000 doses for a relatively common adverse outcome (e.g., myocardial infraction with a background incidence rate of 1400 per 100,000 person years in men older than 85 years old) and more than a million doses are needed to detect a relative risk of 1.5 for a rare adverse outcome (e.g., myocardial infraction with a background incidence rate of 28 per 100,000 person years among those 18-34 years).[73, 74]

Patient and Public Involvement and Engagement (PPIE)

PPIE members (Antony Chuter, Alex Brownrigg and Jillian Beggs) have been involved since the beginning of this project. The research proposal for the Wales analysis has also been reviewed by members of the public. Their contribution includes defining research questions, interpretation, and dissemination of study findings. As part of the ConCOV project [26] in Wales, PPIE members have been involved in the design and evaluation of findings, as well as presentations made to the independent SAIL consumer panel group made up of lay members.

Ethics and dissemination

For Scotland, approvals have been obtained by the National Research Ethics Service Committee (REC), South East Scotland 02 (REC number: 12/SS/0201) and the Public Benefit and Privacy Panel for Health and Social Care (reference number: 1920-0279). For Wales, the data used in this study are available in the SAIL Databank at Swansea University, Swansea, UK. All proposals to use SAIL data are subject to review by an independent Information Governance Review Panel (IGRP). Before any data can be accessed, approval must be given by the IGRP. The IGRP gives careful consideration to each project to ensure proper and appropriate use of SAIL data. When access has been approved, it is gained through a privacy-protecting safe haven and remote access system referred to as the SAIL Gateway. SAIL has established an application process to be followed by anyone who would like to access data via SAIL.[27] Similarly, in Northern Ireland, the project was approved by the HSC Honest Broker Service Governance Board (project number 0064), and accessed through the online secure research platform. Findings will be presented at conferences, published in peer-reviewed journals and to the funders and government COVID-19 advisory bodies as appropriate. Strengthening the Reporting of Observational Studies in Epidemiology (STROBE) and Reporting of studies Conducted using Observational Routinely-collected Data (RECORD) (via the COVID-19 extension) checklists will guide our study findings reporting.[75,76] We will

For peer review only - http://bmjopen.bmj.com/site/about/guidelines.xhtml

also consider using the European Network of Centres for Pharmacoepidemiology and Pharmacovigilance checklist.[77]

Contributors

AS conceived this study and commented on several drafts of the protocol. EV wrote the first draft of the protocol with assistance from TS and SK. SdeL, RT, MJ, DMcG, JW and RH contributed about the English sentinel system and commented on the research methods. DB, DO and SM contributed about the Northern Ireland data and commented on the research methods. AC and JB were involved in the study design and commented on introduction and research methods from a patient and public perspective. DF, CO, AA, SB, GD, LJG, RG, EL, JL, RAL, LN, MP and FT contributed about the Wales data and commented on the research methods. JP contributed on the study design and commented on several drafts. JM, CM, UA, RW, SJS, EM, PH, CRS and CR contributed about the Scotland data and comments on research methods. All authors contributed to the study design. All authors contributed to drafting the protocol. All authors revised the manuscript for important intellectual content. All authors gave final approval of the version to be published.

Acknowledgments

England: Patients and general practices who provide samples and agree to share data with the Oxford-RCGP RSC. EMIS, TPP, In-Practice Systems, Wellbeing software for supporting the RSC data extraction. Public Health England for support and collaboration. Wales: This work uses data provided by patients and collected by the NHS as part of their care and support. We would also like to acknowledge all data providers who make anonymised data available for research. We wish to acknowledge the collaborative partnership that enabled acquisition and access to the de-identified data, which led to this output. The collaboration was led by the Swansea University Health Data Research UK team under the direction of the Welsh Government Technical Advisory Cell (TAC) and includes the following groups and organisations: the Secure Anonymised Information Linkage (SAIL) Databank, Administrative Data Research (ADR) Wales, NHS Wales Informatics Service (NWIS), Public Health Wales, NHS Shared Services Partnership and the Welsh Ambulance Service Trust (WAST). All research conducted has been completed under the permission and approval of the SAIL independent Information Governance Review Panel (IGRP) project number 0911. Northern Ireland: The authors would like to acknowledge the help provided by the staff of the Honest Broker Service (HBS) within the Business Services Organisation Northern Ireland (BSO). The HBS is funded by the BSO and the Department of Health (DoH). The authors alone are responsible for the interpretation of the data and any views or opinions presented are solely those of the author and do not necessarily represent those of the BSO.

Funding

This research is part of the Data and Connectivity National Core Study, led by Health Data Research UK in partnership with the Office for National Statistics and funded by UK Research and Innovation [HDRUK2020.146]. EAVE II is funded by the Medical Research Council [MC_PC_19075] and supported by the Scottish Government. This work is supported by BREATHE - The Health Data Research Hub for Respiratory Health [MC_PC_19004]. BREATHE is funded through the UK Research and Innovation Industrial Strategy Challenge Fund and delivered through Health Data Research UK. ConCOV is supported by the Medical Research Council [MR/V028367/1]; Health Data Research UK [HDR-9006] which receives its funding from the UK Medical Research Council, Engineering and Physical Sciences Research Council, Economic and Social Research Council, Department of Health and Social Care (England), Chief Scientist Office of the Scottish Government Health and Social Care Directorates, Health and Social Care Research and Development Division (Welsh Government), Public Health Agency (Northern Ireland), British Heart Foundation (BHF) and the Wellcome Trust; and Administrative Data Research UK which is funded by the Economic and Social Research Council [grant ES/S007393/1].

Competing interests

AS is a member of the Scottish Government Chief Medical Officer's COVID-19 Advisory Group. RAL reports grants from MRC during the conduct of the study. SJS reports grants from Wellcome Trust, during the conduct of the study; grants from National Institute of Healthcare Research HTA, grants from Tommy's Charity and grants from Chief Scientist for Scotland, outside the submitted work. SdeL is Director of the Royal College of General

For peer review only - http://bmjopen.bmj.com/site/about/guidelines.xhtml

Practitioners Research and Surveillance Centre. He has received grant funding through his University from AstraZeneca, Eli Lilly, GSK MSD, Seqirus, and Takeda. He has been members of advisory boards for AstraZeneca, Sanofi, and Seqirus. DTB is jointly employed by Queen's University Belfast, the Public Health Agency and the Department of Health (Northern Ireland), and he is currently or has been a member of COVID-19 government advisory groups, including the Scientific Advisory Group for Emergencies (SAGE), its subgroups, and the UK Vaccine Effectiveness Expert Panel. All other authors report no conflicts of interest.

REFERENCES

1. World Health Organization. Coronavirus disease (COVID-19) outbreak. Available at: https://www.who.int/emergencies/diseases/novel-coronavirus-2019
2. World Health Organization. Draft landscape of COVID-19 candidate vaccines. Available at: https://www.who.int/publications/m/item/draft-landscape-of-covid-19-candidate-vaccines
3. Regulatory Affairs Professionals Society. COVID-19 vaccine tracker. Available at: https://www.raps.org/news-and-articles/news-articles/2020/3/covid-19-vaccine-tracker
4. European Medicines Agency. COVID-19 vaccines: development, evaluation, approval and monitoring. Available at: https://www.ema.europa.eu/en/human-regulatory/overview/public-health-threats/coronavirus-disease-covid-19/treatments-vaccines/covid-19-vaccines-development-evaluation-approval-monitoring
5. National Health Service. Coronavirus (COVID-19) vaccine. Available at: https://www.nhs.uk/conditions/coronavirus-covid-19/coronavirus-vaccination/coronavirus-vaccine/
6. Polack FP, Thomas SJ, Kitchin N, Absalon J, Gurtman A, Lockhart S, et al. Safety and Efficacy of the BNT162b2 mRNA Covid-19 Vaccine. N Engl J Med. 2020 Dec 31;383(27):2603-2615.
7. Voysey M, Clemens SAC, Madhi SA, Weckx LY, Folegatti PM, Aley PK, et al. Safety and efficacy of the ChAdOx1 nCoV-19 vaccine (AZD1222) against SARS-CoV-2: an interim analysis of four randomised controlled trials in Brazil, South Africa, and the UK. Lancet. 2020 Dec 8:S0140-6736(20)32661-1.
8. FDA Briefing Document. Moderna COVID-19 Vaccine. Available at: https://www.fda.gov/media/144434/download
9. European Medicines Agency. COVID-19 Vaccine AstraZeneca: benefits still outweigh the risks despite possible link to rare blood clots with low blood platelets. Available at: https://www.ema.europa.eu/en/news/covid-19-vaccine-astrazeneca-benefits-still-outweigh-risks-despite-possible-link-rare-blood-clots
10. European Medicines Agency. AstraZeneca's COVID-19 vaccine: EMA finds possible link to very rare cases of unusual blood clots with low blood platelets. Available at: https://www.ema.europa.eu/en/news/astrazenecas-covid-19-vaccine-ema-finds-possible-link-very-rare-cases-unusual-blood-clots-low-blood
11. Public Health England. COVID-19 vaccination and blood clotting. Available at:

For peer review only - http://bmjopen.bmj.com/site/about/guidelines.xhtml

https://www.gov.uk/government/publications/covid-19-vaccination-and-blood-clotting/covid-19-vaccination-and-blood-clotting

12. Diaz GA, Parsons GT, Gering SK, Meier AR, Hutchinson IV, Robicsek A. Myocarditis and Pericarditis After Vaccination for COVID-19. *JAMA.* Published online August 04, 2021. doi:10.1001/jama.2021.13443
13. UK Government. Coronavirus (COVID-19) in the UK. Available at: https://coronavirus.data.gov.uk/details/healthcare#card-people_who_have_received_vaccinations
14. UK Government. Joint Committee on Vaccination and Immunisation: advice on priority groups for COVID-19 vaccination, 30 December 2020. Available at: https://www.gov.uk/government/publications/priority-groups-for-coronavirus-covid-19-vaccination-advice-from-the-jcvi-30-december-2020/joint-committee-on-vaccination-and-immunisation-advice-on-priority-groups-for-covid-19-vaccination-30-december-2020
15. Dhanda S, Osborne V, Lynn E, et al. Postmarketing studies: can they provide a safety net for COVID-19 vaccines in the UK? BMJ Evidence-Based Medicine Published Online First: 21 October 2020. doi: 10.1136/bmjebm-2020-111507
16. Iacobucci G, Mahase E. Covid-19 vaccination: What's the evidence for extending the dosing interval? BMJ. 2021 Jan 6;372:n18.
17. Patel MD, Rosenstrom E, Ivy JS, et al. Association of Simulated COVID-19 Vaccination and Nonpharmaceutical Interventions With Infections, Hospitalizations, and Mortality. *JAMA Netw Open.* 2021;4(6):e2110782.
18. Berlin JA, Glasser SC, Ellenberg SS. Adverse event detection in drug development: recommendations and obligations beyond phase 3. Am J Public Health. 2008;98(8):1366-1371.
19. Robertson E, Reeve KS, Niedzwiedz CL, et al. Predictors of COVID-19 vaccine hesitancy in the UK household longitudinal study. Brain Behav Immun. 2021 May;94:41-50.
20. Madewell ZJ, Yang Y, Longini IM, Halloran ME, Dean NE. Household Transmission of SARS-CoV-2: A Systematic Review and Meta-analysis. JAMA Netw Open. 2020;3(12):e2031756.
21. De Serres G, Skowronski DM, Wu XW, Ambrose CS. The test-negative design: validity, accuracy and precision of vaccine efficacy estimates compared to the gold standard of randomised placebo-controlled clinical trials. Euro Surveill. 2013;18(37):pii=20585.
22. Farrington P, Nash J, Miller E. Case series analysis of adverse reactions to vaccine: a comparative evaluation. Am J Epidem 1996;143(11):1165-73.
23. de Lusignan S, Lopez Bernal J, Zambon M, Akinyemi O, Amirthalingam G, Andrews N, et al. Emergence of a Novel Coronavirus (COVID-19): Protocol for Extending Surveillance Used by the Royal College of General Practitioners Research and Surveillance Centre and Public Health England. JMIR Public Health Surveill. 2020;6(2):e18606.
24. Simpson CR, Robertson C, Vasileiou E, McMenamin J, Gunson R, Ritchie LD, et al. Early Pandemic Evaluation and Enhanced Surveillance of COVID-19 (EAVE II):

For peer review only - http://bmjopen.bmj.com/site/about/guidelines.xhtml

protocol for an observational study using linked Scottish national data. BMJ Open. 2020;10(6):e039097.
25. Swansea University led team secures funding from COVID-19 Rapid Response Call - Population Data Science. Available at: https://popdatasci.swan.ac.uk/swansea-university-led-team-secures-funding-from-covid-19-rapid-response-call/
26. Lyons J, Akbari A, Torabi F, et al. Understanding and responding to COVID-19 in Wales: protocol for a privacy-protecting data platform for enhanced epidemiology and evaluation of interventions. BMJ Open 2020;10:e043010.
27. The Secure Anonymised Information Linkage (SAIL) databank. Available at: https://saildatabank.com/
28. de Lusignan S, Jones N, Dorward J, Byford R, Liyanage H, Briggs J, et al. The Oxford Royal College of General Practitioners Clinical Informatics Digital Hub: Protocol to Develop Extended COVID-19 Surveillance and Trial Platforms. JMIR Public Health Surveill 2020;6(3):e19773.
29. NHS Digital Data Security and Protection Toolkit. Available at: https://www.dsptoolkit.nhs.uk/
30. NHS Digital. Data Security and Protection Toolkit Organisational Search. University of Oxford - Medical Sciences Division – Nuffield Department of Primary Care Health Sciences Organisation code: EE133863-MSD-NDPCHS. Available at: https://www.dsptoolkit.nhs.uk/OrganisationSearch/EE133863-MSD-NDPCHS
31. Public Health England. COVID-19 vaccine surveillance strategy. Available at: https://assets.publishing.service.gov.uk/government/uploads/system/uploads/attachment_data/file/951189/COVID-19_vaccine_surveillance_strategy.pdf
32. de Lusignan S. Codes, classifications, terminologies and nomenclatures: definition, development and application in practice. Inform Prim Care. 2005;13(1):65-70.
33. Oxford-Royal College of General Practitioners (RCGP) Research and Surveillance Centre (RSC). Using Oxford-RCGP RSC for observational studies, surveillance themed dataset. Available at: https://orchid.phc.ox.ac.uk/index.php/orchid-data/
34. de Lusignan S, Williams J. To monitor the COVID-19 pandemic we need better quality primary care data. BJGP Open. 2020;4(2):bjgpopen20X101070.
35. Jani BD, Pell JP, McGagh D, Liyanage H, Kelly D, de Lusignan S, et al. Recording COVID-19 consultations: review of symptoms, risk factors, and proposed SNOMED CT terms. BJGP Open. 2020;4(4):bjgpopen20X101125.
36. de Lusignan S, Liyanage H, McGagh D, Jani BD, Bauwens J, Byford R, et al. COVID-19 Surveillance in a Primary Care Sentinel Network: In-Pandemic Development of an Application Ontology. JMIR Public Health Surveill. 2020 Nov 17;6(4):e21434.
37. COVID-19 vaccination programme in Northern Ireland. Available at: https://www.nidirect.gov.uk/articles/covid-19-vaccination-programme-northern-ireland
38. Turas Vaccination Management Tool. Available at: https://learn.nes.nhs.scot/42708/turas-vaccination-management-tool
39. UK Government. COVID-19 testing data: methodology note. Available at: https://www.gov.uk/government/publications/coronavirus-covid-19-testing-data-methodology/covid-19-testing-data-methodology-note

For peer review only

40. NHS Digital. Data Access Request Service (DARS). Available at: https://digital.nhs.uk/services/data-access-request-service-dars/dars-products-and-services
41. de Lusignan S, Dorward J, Correa A, Jones N, Akinyemi O, Amirthalingam G, et al. Risk factors for SARS-CoV-2 among patients in the Oxford Royal College of General Practitioners Research and Surveillance Centre primary care network: a cross-sectional study. Lancet Infect Dis. 2020;20(9):1034-1042.
42. de Lusignan S, Correa A, Smith GE, Yonova I, Pebody R, Ferreira F, et al. RCGP Research and Surveillance Centre: 50 years' surveillance of influenza, infections, and respiratory conditions. Br J Gen Pract. 2017;67(663):440-441.
43. Department of Health, Northern Ireland. Hospital Statistics. Available at: www.health-ni.gov.uk/articles/inpatient-and-day-case-activity
44. Joy M, Hobbs FDR, McGagh D, Akinyemi O, de Lusignan S. Excess mortality from COVID-19 in an English sentinel network population. Lancet Infect Dis. 2020:S1473-3099(20)30632-0.
45. de Lusignan S, Joy M, Oke J, McGagh D, Nicholson B, Sheppard J, et al. Disparities in the excess risk of mortality in the first wave of COVID-19: Cross sectional study of the English sentinel network. J Infect. 2020;81(5):785-792.
46. Joy M, Hobbs FR, Bernal JL, Sherlock J, Amirthalingam G, McGagh D, et al. Excess mortality in the first COVID pandemic peak: cross-sectional analyses of the impact of age, sex, ethnicity, household size, and long-term conditions in people of known SARS-CoV-2 status in England. Br J Gen Pract. 2020;70(701):e890-e898.
47. NHS Digital. National Health Application and Infrastructure Services (NHAIS). Available at: https://digital.nhs.uk/services/nhais
48. Liyanage H, Williams J, Byford R, de Lusignan S. Ontology to identify pregnant women in electronic health records: primary care sentinel network database study. BMJ Health Care Inform. 2019;26(1):e100013.
49. Liyanage H, Williams J, Byford R, Pathirannehelage S, de Lusignan S. Near-Real Time Monitoring of Vaccine Uptake of Pregnant Women in a Primary Care Sentinel Network: Ontological Case Definition Across Heterogeneous Data Sources. Stud Health Technol Inform. 2019;264:1855-1856.
50. Business Services Organisation. Northern Ireland Maternity System (NIMATS). Available at: http://www.hscbusiness.hscni.net/services/2512.htm
51. Stock SJ, McAllister D, Vasileiou E, et al. COVID-19 in Pregnancy in Scotland (COPS): protocol for an observational study using linked Scottish national data. BMJ Open 2020;10:e042813.
52. Moderna. Available at: https://www.modernatx.com/covid19vaccine-eua/providers/clinical-trial-data
53. European Medicines Agency. Interim guidance on enhanced safety surveillance for seasonal influenza vaccines in the EU. Available at: https://www.ema.europa.eu/en/documents/scientific-guideline/interim-guidance-enhanced-safety-surveillance-seasonal-influenza-vaccines-eu_en.pdf
54. Uptake and comparative safety of new COVID-19 vaccines by age, sex, region, ethnicity, comorbidities, medication, deprivation, risk level and evidence of prior COVID infection. Available at: https://www.qresearch.org/research/approved-research-programs-and-projects/uptake-and-comparative-safety-of-new-covid-19-

For peer review only - http://bmjopen.bmj.com/site/about/guidelines.xhtml

vaccines-by-age-sex-region-ethnicity-comorbidities-medication-deprivation-risk-level-and-evidence-of-prior-covid-infection/
55. National Statistics English indices of deprivation 2019. Available at: https://www.gov.uk/government/statistics/english-indices-of-deprivation-2019
56. de Lusignan S, Sherlock J, Ferreira F, O'Brien S, Joy M. Household presentation of acute gastroenteritis in a primary care sentinel network: retrospective database studies. BMC Public Health. 2020;20(1):445.
57. Welsh Index of Multiple Deprivation 2019. Available at: https://gov.wales/welsh-index-multiple-deprivation-full-index-update-ranks-2019
58. Northern Ireland Multiple Deprivation Measure 2017 (NIMDM2017). Available at: https://www.nisra.gov.uk/statistics/deprivation/northern-ireland-multiple-deprivation-measure-2017-nimdm2017
59. Tippu Z, Correa A, Liyanage H, Burleigh D, McGovern A, Van Vlymen J, et al. Ethnicity Recording in Primary Care Computerised Medical Record Systems: An Ontological Approach. J Innov Health Inform. 2017;23(4):920.
60. Office for National Statistics. Dataset: Lower layer Super Output Area population density (National Statistics), 2020. Available at: https://www.ons.gov.uk/peoplepopulationandcommunity/populationandmigration/populationestimates/datasets/lowersuperoutputareapopulationdensity
61. de Lusignan S, Sherlock J, Akinyemi O, Pebody R, Elliot A, Byford R, et al. Household presentation of influenza and acute respiratory illnesses to a primary care sentinel network: retrospective database studies (2013-2018). BMC Public Health. 2020;20(1):1748.
62. Northern Ireland Statistics & Research agency. Review of the Statistical Classification and Delineation of Settlements. Available at: https://www.nisra.gov.uk/sites/nisra.gov.uk/files/publications/review-of-the-statistical-classification-and-delineation-of-settlements-march-2015%20%281%29.pdf
63. Clift AK, Coupland CAC, Keogh RH, Diaz-Ordaz K, Williamson E, Harrison EM et al. Living risk prediction algorithm (QCOVID) for risk of hospital admission and mortality from coronavirus 19 in adults: national derivation and validation cohort study BMJ 2020; 371:m3731.
64. Fukushima W, Hirota Y. Basic principles of test-negative design in evaluating influenza vaccine effectiveness. Vaccine. 2017;35(36):4796-4800.
65. Public Health England. COVID-19: vaccine surveillance strategy. London, PHE gateway number GW-1763, 2021. Available at: https://www.gov.uk/government/publications/covid-19-vaccine-surveillance-strategy
66. Pebody RG, Whitaker H, Ellis J, Andrews N, Marques DFP, Cottrell S, et al. End of season influenza vaccine effectiveness in primary care in adults and children in the United Kingdom in 2018/19. Vaccine. 2020;38(3):489-497.
67. Pebody R, Djennad A, Ellis J, Andrews N, Marques DFP, Cottrell S, et al. End of season influenza vaccine effectiveness in adults and children in the United Kingdom in 2017/18. Euro Surveill. 2019;24(31):1800488.
68. de Lusignan S, Lopez Bernal J, Byford R, Amirthalingam G, Ferreira F, Akinyemi O, et al. Seasonal influenza surveillance and vaccine effectiveness at a time of co-circulating COVID-19: Oxford-Royal College of General Practitioners (RCGP)

For peer review only - http://bmjopen.bmj.com/site/about/guidelines.xhtml

Research and Surveillance Centre (RSC) and Public Health England (PHE) protocol for winter 2020/21 JMIR Public Health and Surveillance. 08/12/2020:24341 (forthcoming/in press) DOI: 10.2196/24341 URL: https://preprints.jmir.org/preprint/24341

69. Petersen I, Douglas I, Whitaker H. Self controlled case series methods: an alternative to standard epidemiological study designs. BMJ 2016;354:i4515.
70. Benjamini, Y. and Hochberg, Y. Controlling the False Discovery Rate: A Practical and Powerful Approach to Multiple Testing. J R Stat Soc Series B Stat Methodol 1995;57:289-300.
71. Higgins JP, Thompson SG, Deeks JJ, Altman DG. Measuring inconsistency in meta-analyses. BMJ. 2003;327(7414):557-560.
72. Vasileiou E, Simpson CR, Shi T, et al. Interim findings from first-dose mass COVID-19 vaccination roll-out and COVID-19 hospital admissions in Scotland: a national prospective cohort study. Lancet. 2021 May 1;397(10285):1646-1657.
73. Centers for Disease Control and Prevention. Vaccine safety. Rapid Cycle Analysis (RCA) to monitor the safety of COVID-19 vaccines in near real-time within the Vaccine Safety Datalink. Available at: https://www.cdc.gov/vaccinesafety/pdf/VSD-1342-COVID19-RCA-Protocol_FinalV1.1_508.pdf
74. Li X, Ostropolets A, Makadia R, et al. Characterizing the incidence of adverse events of special interest for COVID-19 vaccines across eight countries: a multinational network cohort study. BMJ. 2021;373:n1435.
75. STROBE Statement. Available at: https://www.strobe-statement.org/index.php?id=strobe-home
76. RECORD. Available at: http://www.record-statement.org/
77. European Network of Centres for Pharmacoepidemiology and Pharmacovigilance. Available at: http://www.encepp.eu/index.shtml

For peer review only - http://bmjopen.bmj.com/site/about/guidelines.xhtml

Table 1. Available UK datasets for each data item of interest

Data item	England	Northern Ireland	Scotland	Wales
Exposures				
Pfizer-BioNTech vaccine	GP, NIMS	VMS	GP, TVMT/PHS, SIRS	CVVD
Oxford-AstraZeneca vaccine	GP, NIMS	VMS	GP, TVMT/PHS, SIRS	CVVD
Moderna vaccine	GP, NIMS	VMS	GP, TVMT/PHS, SIRS	CVVD
Laboratory confirmed SARS-CoV-2 infection	GP, Pillar 1&2 SGSS, PHE	Pillar 1 and Pillar 2	ECOSS	PATD (Pillar 1, 2, & 3 data from all NHS and private labs), CVLF testing and results data
Outcomes				
COVID-19 related GP consultation	GP	NA	GP	WLGP
COVID-19 related emergency department consultation	GP, ECDS	Symphony, NIRAES	SMR01	EDDD & EDDS
COVID-19 related hospital admission	GP, SUS	Admissions and discharge dataset	SMR01	PEDW
COVID-19 related ICU admission	GP, CHESS	Admissions and discharge dataset	SICSAG	CDDS, ICCD & ICNC
COVID-19 related death	GP, ONS, SSRS	NHAIS	NRS	ADDE & ADDE (ONS mortality), CDDS & WDSD
Secondary SARS-CoV-2 infection due to household transmission	RCGP RSC household key	Pillar 1 and Pillar 2 dataset	ECOSS	PATD (Pillar 1, 2, & 3 data from all NHS and private labs), CVLF testing and results data
Maternity outcomes	GP, MSDS	NIMATS	COPS study	ADBE (ONS births), MIDS & NCCH
Patient characteristics & confounders				
Age	GP	NHAIS	GP	C19_COHORT20
Sex	GP	NHAIS	GP	C19_COHORT20
Socio-economic status	Post code to IMD	NHAIS	GP	C19_COHORT20
Ethnicity	GP, SUS	VMS	Census 2011	National ethnicity spine (made up of 20 EHR data sources and the ONS Census 2011)
Underlying medical condition	GP	EPD	GP	GP, WLGP, PEDW, CVSP
Type of settlement (urban/rural)	GP	NHAIS	GP	C19_COHORT20
Type of settlement (e.g., private home, care home or social housing)	GP	NHAIS	GP	C19_COHORT20, CARE
Smoking status	GP	NA	GP	WLGP
Body Mass Index (BMI)	GP	NA	GP	WLGP
Prescribed medications	GP	EPD	GP, PIS, HEPMA	WLGP, WDDS

Other non-COVID-19 vaccines (e.g., influenza, pneumococcal)	GP	VMS	GP	WLGP, NCCH
Occupation (e.g., healthcare workers, frontline workers, essential workers)	GP where recorded	Pillar 1 and 2	To be confirmed	HWRA, SWAC
History of healthcare utilisation (e.g., GP consultations, hospital admissions)	GP, SUS	Admissions and discharge dataset	GP, SMR01	PEDW, WLGP

Annual District Birth Extract (ONS Births) (ADBE); Annual District Death Extract (ONS Deaths) (ADDE); COVID Vaccine Data (CVVD); COVID-19 Hospitalisation in England Surveillance System (CHESS); COVID-19 in Pregnancy in Scotland (COPS); COVID-19 Second Generation Surveillance System (SGSS); COVID-19 Shielded People list (CVSP); Critical Care DataSet (CDDS); Electronic Communication of Surveillance in Scotland (ECOSS); Electronic Health Record (HER); Electronic Prescribing Database (EPD); Emergency Care Data Set (ECDS); Emergency Department Data Daily (EDDD); Emergency Department Dataset (EDDS); General Practice (GP); Health and Social Care (HSC) Trusts; Hospital Electronic Prescribing and Medicines Administration (HEPMA); Hospital Episode Statistics (HES); ICNARC – Intensive Care National Audit & Research Centre (All admissions) (ICNC); ICNARC – Intensive Care National Audit & Research Centre (COVID-19 Only admissions) (ICCD); Index of Multiple Deprivation (IMD); Intensive Care Admission (ICU); Intensive Care National Audit & Research Centre (ICNARC); Laboratory Information System (LIS); Maternal Indicators DataSet (MIDS); Maternity Services Dataset (MSDS); National Community Child Health database (NCCH); National Health Applications and Infrastructure Services (NHAIS); National Health Service (NHS); National Immunisation Management Service (NIMS); National Records of Scotland (NRS); Northern Ireland Maternity System (NIMATS); Northern Ireland Regional Accident and Emergency System (NIRAES); Office for National Statistics (ONS); Pathology data COVID-19 Daily (PATD); Patient Episode Database for Wales (PEDW); Prescribing Information System (PIS); Public Health England (PHE); Public Health Scotland (PHS); Royal College of General Practitioners Research and Surveillance Centre (RCGP RSC); Scottish Immunisation & Recall System (SIRS); Scottish Intensive Care Society Audit Group (SICSAG); Scottish Morbidity Record 01 (SMR01); Secondary Users Service (SUS); Turas Vaccination Management Tool (TVMT); Vaccine Management System (VMS); Welsh Demographic Service Dataset (WDSD); Welsh Longitudinal General Practice (WLGP)

For peer review only

Supplementary Material

Appendix 1: Adverse Events Following Immunisation (AEFI) reported in pre-licensure COVID-19 vaccine trials

Adverse event	Pfizer-BioNTech (BNT162b2)	Oxford-AstraZeneca (AZD1222)	Moderna
Abdominal pain		Uncommon	
Acute peripheral facial paralysis (/palsy)	Rare (37 days after dose 1, 3-48 days after dose 2)		Rare (22-32 days after dose 2)
Anaphylaxis*	Not known		Not known
Arthralgia	Very common	Very common	Very common
Chills	Very common	Very common	Very common
Decreased appetite		Uncommon	
Dizziness		Uncommon	
Facial swelling			Rare (1-2 days post-vaccination in vaccinees with history of injection of dermatological fillers)
Fatigue	Very common	Very common	Very common
Headache	Very common	Very common	Very common
Hyperhidrosis		Uncommon	
Hypersensitivity	Not known		Not known
Influenza-like illness		Common	
Injection site bruising**		Very common	
Injection site erythema		Very common	Common
Injection site induration		Common	
Injection site pain	Very common	Very common	Very common
Injection site pruritus	Uncommon	Very common	Uncommon
Injection site rash			Common
Injection site redness	Common		
Injection site swelling	Very common	Very common	Very common
Injection site tenderness		Very common	
Injection site urticaria			Common
Injection site warmth		Very common	
Insomnia	Uncommon		
Lymphadenopathy***	Uncommon	Uncommon	Very common
Malaise	Uncommon	Very common	
Myalgia	Very common	Very common	Very common
Nausea****	Common	Very common	Very common
Pain in extremity	Uncommon		
Pruritus		Uncommon	
Pyrexia*****	Very common	Very common	Very common
Rash		Uncommon	Common
Vomiting		Common	
* Anaphylaxis reported in post-marketing setting for Moderna vaccine.			

For peer review only

** Injection site bruising includes injections site haematoma
*** Lymphadenopathy captured as axillary lymphadenopathy on the same side as the injection site for Moderna vaccine.
**** A higher frequency of pyrexia observed after dose 2 for the Pfizer BioNTech vaccine.
***** Pyrexia includes feverishness
Note. Very common (≥1/10), common (≥1/100 to <1/10), uncommon (≥1/1,000 to <1/100), rare (≥1/10,000 to <1/1,000), very rare (<1/10,000), not known (cannot be estimated).

Very rare events of neuroinflammatory disorders have been reported following vaccination with the Oxford AstraZeneca vaccine, but a causal relationship has not been established.

Appendix 2: List of AEFI for influenza vaccines

- Anaphylactic reactions
- Arthropathy
- Bell's palsy
- Conjunctivitis
- Coryza
- Cough
- Decreased appetite
- Diarrhoea
- Drowsiness
- Epistaxis
- Facial oedema
- Fatigue
- Fever / pyrexia
- Guillain-Barré syndrome
- Headache
- Hoarseness
- Hypersensitivity reactions
- Irritability
- Local symptoms (i.e. local erythema)
- Malaise
- Muscle aches / myalgia
- Nasal congestion
- Nausea
- Oropharyngeal pain
- Peripheral tremor
- Rash
- Rhinorrhoea
- Seizure / febrile convulsions
- Vomiting
- Wheezing

For peer review only - http://bmjopen.bmj.com/site/about/guidelines.xhtml

Appendix 3: Schematic presentation of the self-controlled case series study design

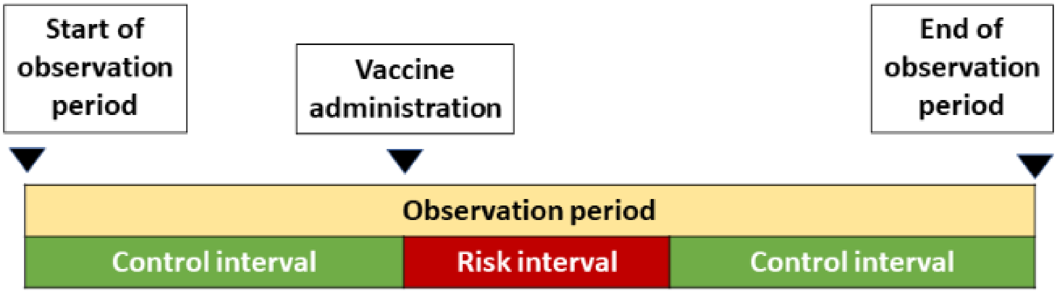

For peer review only

For peer review only - http://bmjopen.bmj.com/site/about/guidelines.xhtml

STROBE Statement—checklist of items that should be included in reports of observational studies

	Item No.	Recommendation	Page No.	Relevant text from manuscript
**Title and abstract**	1	(*a*) Indicate the study's design with a commonly used term in the title or the abstract	2-3	
		(*b*) Provide in the abstract an informative and balanced summary of what was done and what was found	2-3	
**Introduction**				
Background/rationale	2	Explain the scientific background and rationale for the investigation being reported	5-6	
Objectives	3	State specific objectives, including any prespecified hypotheses	6	
**Methods**				
Study design	4	Present key elements of study design early in the paper	6	
Setting	5	Describe the setting, locations, and relevant dates, including periods of recruitment, exposure, follow-up, and data collection	7-12	
Participants	6	(*a*) *Cohort study*—Give the eligibility criteria, and the sources and methods of selection of participants. Describe methods of follow-up<br>*Case-control study*—Give the eligibility criteria, and the sources and methods of case ascertainment and control selection. Give the rationale for the choice of cases and controls<br>*Cross-sectional study*—Give the eligibility criteria, and the sources and methods of selection of participants	13-14	
		(*b*) *Cohort study*—For matched studies, give matching criteria and number of exposed and unexposed<br>*Case-control study*—For matched studies, give matching criteria and the number of controls per case	NA	
Variables	7	Clearly define all outcomes, exposures, predictors, potential confounders, and effect modifiers. Give diagnostic criteria, if applicable	10-12	
Data sources/ measurement	8*	For each variable of interest, give sources of data and details of methods of assessment (measurement). Describe comparability of assessment methods if there is more than one group	7-10	
Bias	9	Describe any efforts to address potential sources of bias	11-14	
Study size	10	Explain how the study size was arrived at	15	

Continued on next page

For peer review only - http://bmjopen.bmj.com/site/about/guidelines.xhtml
For peer review only

Quantitative variables	11	Explain how quantitative variables were handled in the analyses. If applicable, describe which groupings were chosen and why	10-12
Statistical methods	12	(*a*) Describe all statistical methods, including those used to control for confounding	13-15
		(*b*) Describe any methods used to examine subgroups and interactions	13-15
		(*c*) Explain how missing data were addressed	13
		(*d*) *Cohort study*—If applicable, explain how loss to follow-up was addressed<br>*Case-control study*—If applicable, explain how matching of cases and controls was addressed<br>*Cross-sectional study*—If applicable, describe analytical methods taking account of sampling strategy	13-15
		(*e*) Describe any sensitivity analyses	13-15
**Results**			
Participants	13*	(a) Report numbers of individuals at each stage of study—eg numbers potentially eligible, examined for eligibility, confirmed eligible, included in the study, completing follow-up, and analysed	NA
		(b) Give reasons for non-participation at each stage	NA
		(c) Consider use of a flow diagram	NA
Descriptive data	14*	(a) Give characteristics of study participants (eg demographic, clinical, social) and information on exposures and potential confounders	NA
		(b) Indicate number of participants with missing data for each variable of interest	NA
		(c) *Cohort study*—Summarise follow-up time (eg, average and total amount)	NA
Outcome data	15*	*Cohort study*—Report numbers of outcome events or summary measures over time	NA
		Case-control study—Report numbers in each exposure category, or summary measures of exposure	NA
		Cross-sectional study—Report numbers of outcome events or summary measures	NA
Main results	16	(*a*) Give unadjusted estimates and, if applicable, confounder-adjusted estimates and their precision (eg, 95% confidence interval). Make clear which confounders were adjusted for and why they were included	NA
		(*b*) Report category boundaries when continuous variables were categorized	NA
		(*c*) If relevant, consider translating estimates of relative risk into absolute risk for a meaningful time period	NA

Continued on next page

For peer review only

Other analyses	17	Report other analyses done—eg analyses of subgroups and interactions, and sensitivity analyses	NA
**Discussion**			
Key results	18	Summarise key results with reference to study objectives	NA
Limitations	19	Discuss limitations of the study, taking into account sources of potential bias or imprecision. Discuss both direction and magnitude of any potential bias	NA
Interpretation	20	Give a cautious overall interpretation of results considering objectives, limitations, multiplicity of analyses, results from similar studies, and other relevant evidence	NA
Generalisability	21	Discuss the generalisability (external validity) of the study results	NA
**Other information**			
Funding	22	Give the source of funding and the role of the funders for the present study and, if applicable, for the original study on which the present article is based	16

*Give information separately for cases and controls in case-control studies and, if applicable, for exposed and unexposed groups in cohort and cross-sectional studies.

Note: An Explanation and Elaboration article discusses each checklist item and gives methodological background and published examples of transparent reporting. The STROBE checklist is best used in conjunction with this article (freely available on the Web sites of PLoS Medicine at http://www.plosmedicine.org/, Annals of Internal Medicine at http://www.annals.org/, and Epidemiology at http://www.epidem.com/). Information on the STROBE Initiative is available at www.strobe-statement.org.

For peer review only
